# Language Learning as a Non-Pharmacological Intervention in Older Adults with (Past) Depression

**DOI:** 10.3390/brainsci15090991

**Published:** 2025-09-15

**Authors:** Jelle Brouwer, Floor van den Berg, Remco Knooihuizen, Hanneke Loerts, Merel Keijzer

**Affiliations:** 1 Center for Language and Cognition Groningen, University of Groningen, 9712 EK Groningen, The Netherlandsr.m.knooihuizen@rug.nl (R.K.);; 2 Center for Language Studies, Radboud University, 6526 HT Nijmegen, The Netherlands; 3 Amsterdam Center for Language and Communication, University of Amsterdam, 1000 BP Amsterdam, The Netherlands

**Keywords:** late life depression, older adults, language learning, intervention

## Abstract

**Background:** Language learning has been proposed as a non-pharmacological intervention to promote healthy aging. This intervention has not been studied in older adults with a history of depression, who experience high prevalence of cognitive dysfunction. This small-scale study was the first to investigate the potential efficacy of language learning in older adults with (past) depression. **Methods:** Data on psychosocial well-being, cognitive functioning, and language outcomes were collected in nineteen participants with (past) depression (M = 69.7 years old, SD = 2.9; 79% women, 21% men) and a control group of fifteen older adults without depression in the past 25 years (M = 70.1 years old, SD = 3.8; 60% women, 40% men). Data were collected before, immediately after, and four months after completing a three-month language course. **Results:** Participants with (past) depression showed significant decreases in apathy, social loneliness, and cognitive failures, and increases in associative memory and global cognition. The control group improved on associative memory and letter-number sequencing. Both groups improved in linguistic self-confidence and lexical access to English, while the group with (past) depression also improved on listening and speaking proficiency. **Conclusions:** The intervention had limited benefits for cognition and psychosocial well-being, but (longer) group-based learning interventions may build up social and motivational reserves protecting against morbidity. Research with larger samples and a no-training control sample is needed to further support these findings.

## 1. Introduction

Major depressive disorder after the age of 60 is a prevalent illness [1]. Aside from affective and somatic symptoms, lower cognitive functioning is commonly reported in older adults with depression [2] and these cognitive symptoms often persist after depression remission [3]. Furthermore, the occurrence of depression in midlife and later in life has been identified both as a risk factor for [4] and a prodromal symptom of dementia [3]. Several types of interventions to potentially increase well-being and cognitive functioning have been investigated both in populations with and without depression. One of the most commonly studied interventions is computerized cognitive training, whereby participants train cognitively demanding tasks [5]. Although study designs vary, this method has been criticized for having too much overlap between the games used in training and the outcome measures, making it difficult to distinguish intervention gains from retest effects, and for not addressing underlying factors that contribute to depression, like social isolation [5]. Clearer transfer effects have been found in other non-pharmacological interventions such as group-based physical exercise [6], showing that improvement in cognitive functions is possible; however, research is scarce, and results regarding both a change in depressive symptoms and cognitive functioning are inconclusive [7]. Therefore, additional interventions should be explored.

This past decade, language learning has received special attention as an intervention that can potentially improve both well-being [8] and cognitive functioning [9]. Lifelong bilingualism has been associated with higher levels of cognitive reserve in older adulthood [10], which is linked to preserved cognitive functioning in older age due to an increased resilience against brain atrophy [11]. As such, lifelong bilingualism has been associated with a later onset of dementia symptoms [12]. These protective effects are thought to stem from the regulation of the constant co-activation of known languages. That is, when speaking one language, all other known languages are active to some extent [13]; the activation of non-target languages must be suppressed to prevent unwanted interference. This constant ‘juggling’ of control involves certain executive functions (e.g., inhibition, selection) that fall under the umbrella of attentional control [14]. These processes already occur in early stages of language learning [15]. This has led researchers to investigate later-life language learning as a tool for healthy aging, not least because the same neural networks that decline in aging have been hypothesized to be strengthened during language learning later in life [9].

While comparatively little focus has been placed on outcomes related to well-being, like reduced loneliness and depression, studies have found later-life language learning to positively impact healthy older adults: language learning has been called an enjoyable experience [8,16], which contributes to one’s sense of autonomy [17] and social well-being [18]. However, most studies did not use validated tools to assess indices of well-being, meaning that it is difficult to assess if later-life language learning could lead to clinically relevant improvements in well-being. With regards to cognitive functioning, some studies find evidence for cognitive adaptations [16,17,19] while other studies do not [20,21,22]. Notably, however, lower baseline levels of cognitive functioning have been associated with larger improvements in older language learners [23], and studies in older adults with Mild Cognitive Impairment (MCI) have also found significant cognitive improvements following a language course [24].

No work on later-life language learning to date has investigated people with (past) depression. Instead, most studies have investigated cognitively healthy older adults, who score near-ceiling on well-being and neuropsychological tests [25]. The evidence discussed above, however, suggests that language learning can be both a cognitively and socially stimulating activity, which may be especially advantageous for people with lower levels of baseline cognitive functioning, including older adults with a current or past episode of depression [3]. This study is the first to investigate the effect of later-life language learning on cognitive functioning and well-being in older adults with a current depressive episode or a history of depression. We expect baseline levels of cognitive functioning and well-being to be lower in participants with (past) depression in comparison to a control group without mood complaints. Furthermore, we hypothesize that older adults with (past) depression will show larger improvements on indices of cognitive functioning and well-being than the control group.

## 2. Method

### 2.1. Study Design

In this intervention study, the efficacy of a three-month English language learning intervention was assessed in two independent samples (participants with (past) depression and a control group without mood complaints in the past 25 years) recruited in the Netherlands. Recruitment and interventions took place from February 2021 to July 2022. Data on cognitive functioning and well-being were collected before, immediately after, and four months after completion of the intervention. Data collection and the interventions were conducted through videoconferencing due to COVID-19. This study was part of a larger project that also comprised a randomized-controlled trial (RCT) comparing effects of a language course with those of a music course and lecture series in older adults not experiencing cognitive decline or depression [26]. The language sample of this RCT was used as the control group for participants with (past) depression in this study.

### 2.2. Participants and Data Collection Procedures

Two samples of community-dwelling older adults (aged ≥ 65; range = 65–76) in the Netherlands were included, both of whom were enrolled in the language intervention. This study did not include a no-intervention control group. This choice was an ethical consideration out of concern for the welfare of the participants (see article 1.1 in the core principles of the Canadian panel of research ethics). We expected participants to enroll in the study because of a personal interest in (language) learning. Being randomized into a no-training control group, then, may have been disappointing and disempowering to participants, which in turn may have negatively affected their well-being. This consideration was endorsed by the Ethical Research Committee (CETO) of the University of Groningen. Since the researchers had no formal clinical background to address these potential negative feelings, we opted for the current design (see Section 4.1. for more about this limitation). The experimental group (*n* = 19) had (past) depression (major depression ≤ 25 years ago based on DSM-5 criteria) or current depressive symptoms (DSM-5 criteria for major depression and/or a Geriatric Depression Scale score ≥ 5). The control group (*n* = 15) had been free of depression or other psychiatric disorders in the past 25 years. Other inclusion criteria were speaking Dutch ≥ 80% in their daily lives, having normal intelligence [27], being able to perform instrumental activities of daily living [28], and having access to a laptop or desktop computer with a webcam and microphone.

Exclusion criteria were: English proficiency ≥ B2 on the CEFR scale, Montreal Cognitive Assessment (MoCA) score ≤ 22 [29], playing a musical instrument ≥ once a week in the past 20 years, severe reading difficulties, neurological problems, uncorrected visual or hearing impairment, past or present alcohol and/or drug dependency, and using benzodiazepines ≥ 3 times per week. Due to difficulties recruiting suitable participants, the criterion for benzodiazepine use was loosened during recruitment (where it used to be no benzodiazepines), as was the criterion for presence of depression (this was initially only ≥5 GDS or current major depressive episode).

Participants were mostly recruited through the local library network Biblionet and participant repository Hersenonderzoek.nl. After participants provided verbal informed consent, a brief telephone screening determined preliminary inclusion. In case of a positive evaluation, written informed consent was obtained. Subsequently, full eligibility was assessed in a screening appointment held via videoconferencing.

Data were collected through videoconferencing with the participant and an experimenter in two test sessions lasting between 1 and 1.5 h each. Sessions typically took place on the same day, with a 2.5 h break in between sessions. English proficiency was assessed before and after the intervention in a separate testing session taking approximately 45 min.

The study protocol was approved by the ethics committee of the Faculty of Arts at the University of Groningen (ref. nr.: 6989509), and was executed in accordance with the Declaration of Helsinki.

### 2.3. Outcome Variables and Covariates

A battery of questionnaires and tasks, (adapted from [30], also used in [26]) was used to measure cognitive functioning, psychosocial well-being, and English proficiency. Table 1 gives an overview of all the outcome measures.

### 2.4. English Course

Participants followed a three-month English course consisting of one introductory class (explaining the course’s goals and materials) and six bi-weekly classes led by an experienced instructor. Classes, taught through videoconferencing, lasted approximately 1.5 h each. Classes focused mostly on communicating in English. Additionally, participants were asked to engage in self-study for five days a week, 45 min a day, using the materials and online environment of the Leidse Onderwijsinstellingen (LOI), a Dutch commercial provider of self-study courses. A participant adhering to the study protocol would spend approximately 4.5 h per week undertaking activities related to the intervention (e.g., going to class, self-study). Adherence to the self-study protocol was self-reported in digital study diaries that were sent out every week.

### 2.5. Analysis

All analyses were conducted in R version 4.2.0 [44]. All dependent variables (DVs) were normalized (z-scaled) and centered to ease interpretation. Scores for some DVs were reversed, so an increased score always represented an improvement. A single multivariate multiple regression model was built for cognitive and psychosocial DVs, to reduce Type I errors [45]. A second model assessed if differentiating between participants with current (in the past six months) and past (previous 25 years to six months) depressive symptoms improved the model fit. Since this was not the case, we continued the analysis without distinguishing between those with current and past depression. Language outcomes were analyzed in a separate model, as they were only assessed at pre-test and post-test. Models included covariates for age, sex, cognitive reserve index score [46] and premorbid intelligence [27], and a random intercept per participant. No power analysis was conducted beforehand.

Planned comparisons and effect sizes (Cohen’s d) were calculated using ‘emmeans’ version 1.8.4.-1 [47]. Results were visualized using ‘interactions’ version 1.1.5 [48]. Model assumptions were checked using ‘performance’ version 0.10.2. [49]. Some of the categorical variables and some of the interaction terms between categorical variables had high variance inflation factor values (VIFs), suggesting multicollinearity (see Supplementary S1). We ran the model again without interaction terms, which yielded no problematic VIFs. This suggests that the high VIF scores stem from the inclusion of interaction terms, which is a condition under which multicollinearity can be safely ignored [50].

## 3. Results

### 3.1. Sample Characteristics

A total of 37 participants were included in the language intervention (for details on recruitment and exclusion, please see Supplementary S2). All participants in the control group completed the entire intervention. Two participants with (past) depression dropped out during the course due to stress, one did not complete the post-test measurement due to illness, and one participant did not respond to invitations for the follow-up assessment. The final sample consisted of 34 participants: 15 in the control group and 19 in the (past) depression group. Demographic baseline characteristics stratified by group are listed in Table 2. As expected, the control group had higher global cognitive functioning and higher levels of cognitive reserve than those with (past depression) at screening. On average, based on the time spent on the intervention each week participants in both groups adhered to the protocol.

### 3.2. Intervention Outcomes

**Figure 1:**
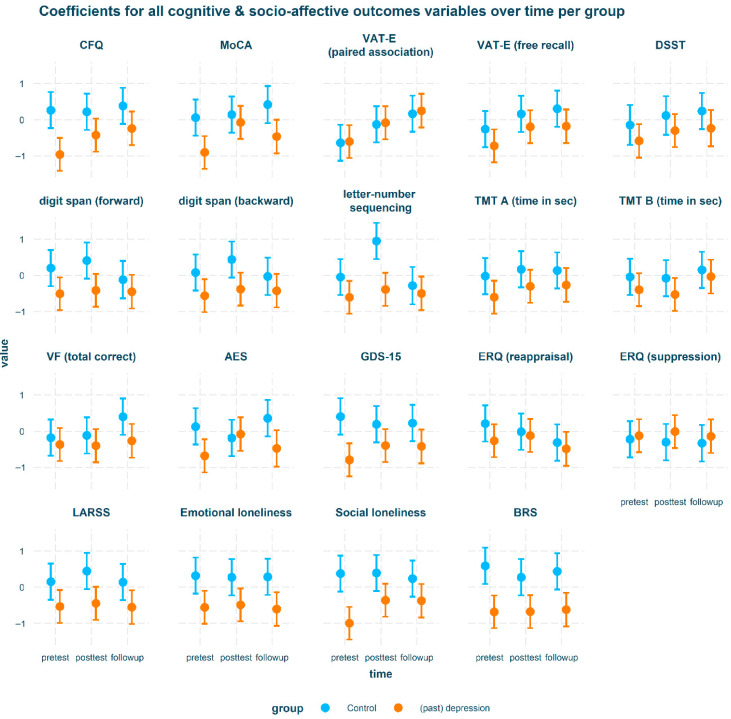
Intervention outcomes for all cognitive and psychosocial variables stratified by group.

**Figure 2:**
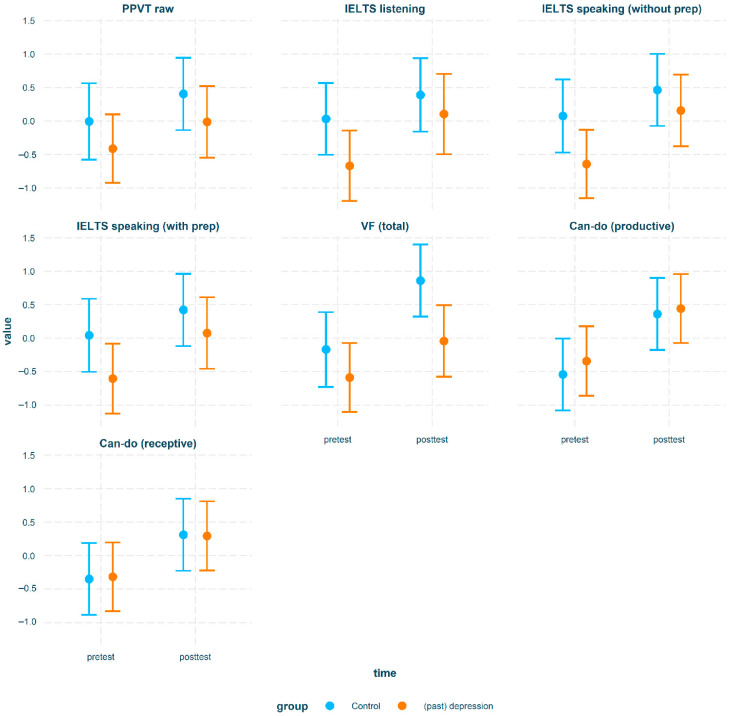
Intervention outcomes for all English proficiency variables stratified by group.

Table 3 shows the raw data stratified by time and group. Table 4 shows significant results from our planned comparisons. Cognitive and psychosocial intervention outcomes are visualized in Figure 1 and English outcomes in Figure 2. Only significant results will be discussed below. Model building procedures and complete results (including non-significant findings) are available in Supplementary S1 and S3. 

#### 3.2.1. Association of Language Learning with Psychosocial Measures

**Between-group differences**

Please note that AES, GDS-15, LARSS, and loneliness were reversed for the analyses. At pre-test, post-test, and follow-up, the control group scored significantly higher than those with (past) depression on LARSS, emotional loneliness, and BRS. Additionally, the control group had higher GDS-15 scores at baseline, higher AES scores at pre-test and follow-up, and higher social loneliness scores at pre-test and post-test.

**Within-group changes over time**

Those with (past) depression improved significantly from pre-test to post-test on AES and social loneliness. This improvement was maintained at follow-up.

**Between-group differences in change over time**

There was a significant difference in the change from pre-test to post-test between groups on the AES, such that apathy levels decreased in participants with (past) depression while the control group’s apathy levels increased. This pattern reversed from post-test to follow-up, and the difference in change over time between the two groups was again significant.

#### 3.2.2. Association of Language Learning with Cognitive Functioning

**Between-group differences**

At pre-test, the control group scored significantly higher than those with (past) depression on CFQ, MoCA, and digit span forward. At post-test, the control group scored higher on CFQ, digit span forward, digit span backward, and letter-number sequencing. At follow-up, the control group scored higher on the MoCA and verbal fluency task.

**Within-group change over time**

Participants with (past) depression improved significantly from pre-test to post-test on the MoCA. This group also showed a significant improvement between pre-test and follow-up for CFQ and VAT-E paired association. Participants in the control group improved significantly from pre-test to post-test on letter-number sequencing, which significantly decreased again from post-test to follow-up. This group also improved on the VAT-E paired association task from pre-test to follow-up.

**Between-group differences in change over time**

The control group showed a significantly larger improvement from pre-test to pos-test than participants with (past) depression on letter-number sequencing. The subsequent decrease from post-test to follow-up was significantly larger in the control group than in participants with (past depression).

### 3.3. Associations of Language Learning with English Proficiency

At pre-test, the control group scored significantly higher than those with (past) depression on all IELTS measures. At post-test, the control group only scored significantly higher than those with (past) depression on the English verbal fluency task.

**Within-group changes over time**

Those with (past) depression improved significantly from pre-test to post-test on all IELTS measures, English verbal fluency, self-rated English productive skills, and self-rated English productive skills. The control group improved significantly on English verbal fluency, self-rated productive English skills, and self-rated receptive English skills.

**Between-group differences in change over time**

There were no significant differences in change in any of the English proficiency measures across time-points between groups.

## 4. Discussion

This small-scale study investigated if later-life language learning stimulates psychosocial well-being and cognitive functioning in older adults with (past) depression and a control group without depression in the last 25 years. For psychosocial well-being, we found a reduction in apathy levels from pre-test to post-test only in participants with (past) depression, suggesting that the course rekindled their overall motivation and goal-directed behavior. Apathy scores increased again at follow-up, suggesting that continued course engagement is needed to retain improvements. Furthermore, while participants with (past) depression reported higher levels of loneliness than the control group across time-points, we found a sustained reduction in social loneliness in participants with (past) depression, meaning they felt more satisfied with their broader social networks [51]. Contrary to our findings, Ware et al. [22] found no change in loneliness after a language course. Potentially this discrepancy arises because the earlier study did not differentiate between social and emotional loneliness. We argue it is necessary to do so, as one cannot necessarily expect intimate relationships, which the emotional loneliness subscale taps into, after a short course. The reduced social loneliness we report is promising, as low levels of loneliness are often associated with reduced overall morbidity and mortality [52], while high levels of loneliness have been associated with increased depression severity at two-year follow-up [53]. Likewise, reduced apathy could also be positive in the longer term, as high apathy levels in late-life depression are associated with increased mortality [54] and higher levels of depression at six-year follow-up [55]. We speculate that language learning (or other group-based learning interventions) could build up a social and motivational reserve over time [56], subsequently reducing morbidity in the long-term. To assess this, future studies should include longer follow-up periods. Furthermore, future work should investigate if the reduced apathy and social loneliness associated with group-based language learning could provide additive benefits when coupled with methods that, contrary to language learning, are effective for targeting depressive symptoms, resilience, and emotion regulation, such as cognitive behavioral therapy [57].

In line with some previous studies conducted with participants without depression [19,20,21], most cognitive outcome measures did not change from pre- to post-test in the control group. Participants with (past) depression, on the other hand, improved significantly on the MoCA, a screening test for cognitive impairment, from pre-test to post-test, but not on tasks that appear in the MoCA in a shortened or modified form (VF, TMT, digit-span). This is partially in line with previous work, which postulated that specifically performance on comprehensive measurements of cognitive functioning should improve, since language learning activates extensive brain networks [19]. However, the control group did not significantly improve on this measure, potentially because they scored closer to ceiling level at baseline. Significant reductions in self-assessed cognitive failures were only reported in participants with (past) depression. Past research has reported that subjective cognitive complaints are more strongly linked to depressive symptoms than to actual cognitive functioning [58], suggesting that participants with (past) depression were more sensitive to improvements on the cognitive failures questionnaire. A surprising result was the significant improvement of letter-number sequencing in the control group, while no such improvement was found in the (past) depression group. While we do not have a cogent explanation, we speculate that the higher baseline levels of English proficiency in the control group meant that they more easily re-trained working-memory manipulation processes needed in speaking additional languages. That is, when speaking a second language, one needs to keep the intended message (i.e., the words themselves) in working memory, while simultaneously applying a word order that is different from one’s first language. Perhaps the higher levels of English proficiency found in the control group at baseline meant they more easily retrained these processes of manipulating items held in working memory (which letter-number sequencing indexes). Lastly, both groups showed an identical improvement on associative memory from pre-test to follow-up. Potentially, the skill of mapping new vocabulary in an additional language to existing semantic concepts, as undertaken in language learning, led to delayed transfer effects on the VAT-E. However, this effect only became significant at follow-up and only one version of this test was used, which taken together suggests a test–retest effect. Notably, our study differs from some later-life language learning interventions, as our participants had at least some basis in the taught language. Future research should include both completely unknown languages and languages for which participants have some basis, to see if this leads to differential effects in those with (past) depression.

The linguistic outcome measures paint a clearer picture. A short but intense language course led to significant improvements in speaking and listening proficiency (IELTS), lexical access, and linguistic self-confidence in participants with (past) depression. This is in line with previous research with older adults without depression, which also reported significant improvements in proficiency after or within three months of individual and classroom-based training [59,60]. The control group in our study also showed improvements in lexical access and linguistic self-confidence, but not on IELTS, presumably because their English proficiency was higher at baseline. These improvements in linguistic skills and self-confidence are positive, as seeing oneself improve may positively affect one’s overall motivation and self-efficacy [17,22]. Future research could investigate the link between linguistic self-confidence and overall self-efficacy in more detail.

### 4.1. Strengths and Weaknesses

A strength of the study was that it focused on psychosocial, cognitive, as well as linguistic factors, providing a more holistic picture of the effects of later-life language learning. Another strength was including a follow-up period, shedding light on whether continued course engagement may be necessary for retainment. Furthermore, we screened and controlled for potentially confounding variables. Lastly, our study expanded on previous work by including validated tools used in clinical settings and by focusing on participants with (past) depression, a group which has not been represented yet in the later-life language learning field, even though they could benefit from this intervention.

A major limitation of the study is that it is underpowered due to the small sample size. While the call for participants was distributed to a large participant database, including a large sample of people with (past) depression, very few older adults experiencing a current major depressive episode enrolled. This is a common issue with depression trials, potentially caused by participants viewing the study as incurring a high burden or presenting a risk of symptom exacerbation [61]. The small sample overall could be explained by relatively stringent inclusion criteria regarding participants’ language background (cf. [26]). This small sample means that the obtained results must be interpreted with caution. A further limitation to the robustness of our findings was not including a no-training control group (but see the method section for the ethical consideration underlying this design choice). The lack of a group that did not receive an intervention means that it is [25] not possible to fully attribute changes in cognition and psychosocial well-being to the intervention itself. Instead, other environmental factors or test–retest effects may have played a role in the results obtained for the two respective samples recruited for this study. Since our design cannot rule out these factors, caution must be applied when interpreting the results. Future research should include samples that do not take part in an intervention to make the results more robust. Lastly, the study protocol was conducted during the COVID-19 pandemic, meaning that both the intervention and the data collection were carried out via the internet. As a result, participation was limited to those with pre-existing proficiency in using computers. Furthermore, data collection via videoconferencing meant that participants were not always in an environment without distractors. However, digital data-collection also had its strengths, as it allowed older adults with reduced mobility to participate.

## 5. Conclusions

This small-scale study explored the effects of a three-month language learning intervention on psychosocial well-being, cognitive functioning, and English proficiency in older adults with (past) depression and a control group. Overall, language learning seems to have limited immediate benefits for psychosocial well-being and cognition. However, indicators of psychosocial well-being included in this study suggest that a social and motivational reserve may be built up in the process of following a group-based intervention. Furthermore, we report increases in linguistic self-confidence in both groups, which may also be associated with higher levels of self-efficacy and motivation in the long term.

## Figures and Tables

**Table 1:** Overview of outcome measures, as also reported in [26].

(Sub) Domain	Measure	Reference	Primary Outcome
**Cognition**			
Cognitive flexibility	Trail Making Test B (TMT-B)	[31]	Seconds to complete
	Wechsler Adult Intelligence Scale (WAIS-IV) digit-symbol substitution	[32]	Number of symbols in 120 s (0–133)
	WAIS-IV letter-number sequencing (Please note:. Also indexes working memory)	[32]	Total correctly recalled sequences (0–21)
Episodic memory	Visual Association Task-Extended (VAT-E) paired association subscale	[33]	Number of correctly recalled images (1–24)
	VAT-E free recall subscale	[33]	Number of correctly recalled images (1–48)
Global cognition	Montreal Cognitive Assessment (MoCA)	[34]	Overall score (0–30)
Subjective cognitive functioning	Cognitive Failures Questionnaire (CFQ)	[35]	Degree of experienced cognitive failures (0–100)
Attention/processing speed	Trail Making Task A (TMT-A)	[31]	Seconds to complete
Working memory	WAIS-IV digit span forward	[32]	Total correctly recalled sequences (0–12)
	WAIS-IV digit span backward	[32]	Total correctly recalled sequences (0–12)
Executive functioning/verbal fluency	Phonetic verbal fluency (DAT, KOM, or PGR)		Total correctly named words
**Psychosocial well-being**			
Depression symptoms	Geriatric Depression Scale 15-item version (GDS-15)	[36]	Depression severity (0–15)
Psychiatric disorders	Structured Clinical Interview for the DSM-V (SCID-5)	[37]	Presence of current or past psychiatric disorders
Loneliness	De Jong-Gierveld 6-item loneliness scale	[38]	Social and emotional loneliness (0–3)
Resilience	Brief Resilience Scale	Original: [39] Translation: [40]	Ability to bounce back after setbacks and stressful life events (1–5)
Apathy	Apathy Evaluation Scale (AES)	[41]	Total score (18–72)
Rumination	Leuven Adaptation of the Rumination on Sadness	[42]	Total score (21–105)
**English proficiency**			
Lexical access	English Phonetic Verbal Fluency (PRW, CFL, FAS)		Total correctly named words
Vocabulary size	Peabody Picture Vocabulary Naming Test (PPVT-4)	[43]	Raw score (ceiling item minus number of incorrect answers) (0–228)
Speaking proficiency	International English Language Testing System (IELTS) speaking test		Overall score on spontaneous (0–9) and prepared speech (0–9)
Listening proficiency	International English Language Testing System (IELTS) listening test		Overall summed score on listening tests (0–20)

**Table 2:** Baseline characteristics of participants who completed the intervention stratified by group.

	Control Group Not Depressed in the Past 25 Years or Longer (*n* = 15)	Participants with Current Depression or Depression in the Past 25 Years (*n* = 19)	*p*
	M (SD)/*n* %	M (SD)/*n* %	
Age (years)	70.1 (3.8)	69.7 (2.9)	n.s.
Gender Women Men	9 (60%) 5 (40%)	15 (79%) 4 (21%)	n.s.
Current working situation	Retired: 11 (73.3%) Volunteering: 1 (6.7%) Part-time: 1 (6.7%) Informal care: 1 (6.7%) Self-employed: 1 (6.7%)	Retired 17: (89.5%) Volunteering: 1 (5.3%) Part-time: 1 (5.3%)	n.s.
MoCA (at screening)	26.9 (1.2)	25.1 (2.4)	<0.05 *
Cognitive reserve	144.7 (19.7)	131.2 (13.5)	<0.05 *
Premorbid intelligence	92.5 (8.5)	94.0 (8.2)	n.s.
Number of participants with GDS ≥ 5 OR current major depressive disorder according to DSM-V criteria at screening	0	9	<0.01 **
Average number of hours per week spent on intervention (4.5 h requested by researchers)	5.7 (4.3)	5.2 (2)	

Please note: Baseline characteristics were compared using Fisher’s exact test for ategorical data, and Mann–Whitney U tests for continuous data. n.s.: not significant; * *p* < 0.05; ** *p* < 0.01.

**Table 3:** Raw data stratified by time and group.

	Control Group (*n* = 15)			Participants with Current or Past Depression (*n* = 19)		
**Cognitive** **Measures**	**Pretest**	**Posttest**	**Followup**	**Pretest**	**Posttest**	**Followup**
CFQ	31.40 (11.68)	32.20 (8.13)	30.33 (10.41)	46.11 (13.43)	39.89 (8.37)	37.56 (12.21)
MoCA	26.93 (1.87)	27.07 (2.25)	27.71 (1.14)	24.95 (2.46)	26.63 (1.67)	25.83 (1.69)
VAT-E (paired association)	14.33 (4.82)	17.33 (6.26)	19.07 (5.81)	14.58 (6.21)	17.63 (6.95)	19.72 (4.96)
VAT-E (free recall)	24.93 (7.52)	28.53 (9.36)	29.67 (10.33)	20.89 (8.03)	25.42 (7.81)	25.61 (8.68)
DSST	60.67 (7.52)	63.38 (9.12)	64.67 (9.42)	54.06 (14.98)	57.68 (17.58)	58.33 (13.96)
Digit span forward	7.20 (2.18)	7.60 (2.69)	6.57 (2.03)	5.74 (1.41)	5.89 (1.73)	5.83 (2.04)
Digit span backward	7.60 (1.92)	8.27 (2.31)	7.43 (1.79)	6.37 (1.38)	6.68 (1.63)	6.61 (2.20)
Letter-number sequencing	10.93 (2.87)	13.53 (3.56)	10.36 (1.86)	9.47 (1.84)	10.00 (2.00)	9.72 (1.71)
TMT-A	38.73 (8.22)	36.93 (6.49)	37.40 (7.83)	44.79 (12.49)	41.84 (12.02)	41.39 (12.22)
TMT-B	78.53 (19.46)	79.93 (26.33)	74.53 (19.71)	87.26 (24.49)	90.95 (35.68)	78.50 (16.22)
Dutch verbal fluency	46.53 (9.66)	47.00 (9.65)	52.40 (12.00)	44.58 (10.30)	44.05 (11.79)	45.56 (9.92)
**Psychosocial well-being**	**Pretest**	**Posttest**	**Followup**	**Pretest**	**Posttest**	**Followup**
AES	26.2 (3.86)	28.2 (5.39)	25.0 (4.90)	31.0 (6.77)	27.5 (5.59)	30.0 (7.25)
GDS-15	0.40 (1.30)	1.13 (2.56)	1.07 (2.81)	4.21 (3.55)	3.00 (3.46)	3.06 (3.24)
ERQ (cognitive reappraisal)	28.27 (7.27)	26.53 (8.87)	24.33 (8.57)	24.95 (5.03)	25.84 (5.89)	23.28 (7.16)
ERQ (expressive suppression)	13.00 (4.11)	13.47 (4.16)	13.67 (6.85)	12.47 (5.33)	12.00 (5.12)	12.61 (4.17)
LARSS	32.67 (14.52)	28.27 (15.41)	33.33 (16.47)	43.58 (14.49)	42.47 (14.01)	44.06 (16.46)
Emotional loneliness	0.20 (0.77)	0.27 (0.80)	0.27 (0.80)	1.11 (1.15)	1.05 (1.08)	1.17 (1.04)
Social loneliness	0.27 (0.59)	0.27 (0.80)	0.47 (1.06)	1.89 (1.33)	1.16 (0.90)	1.17 (1.34)
BRS	3.91 (0.43)	3.69 (0.69)	3.79 (0.62)	3.08 (0.55)	3.07 (0.51)	3.11 (0.56)
**Language outcomes**	**Pretest**	**Posttest**	**Followup**	**Pretest**	**Posttest**	**Followup**
English verbal fluency	26.62 (5.77)	36.27 (11.18)	N/A	23.47 (8.02)	28.69 (7.17)	N/A
PPVT	153.25 (17.72)	160.60 (15.01)	N/A	144.32 (25.93)	153.44 (25.30)	N/A
IELTS speaking (without preparation)	5.07 (1.21)	5.53 (1.46)	N/A	4.16 (1.34)	5.25 (1.00)	N/A
IELTS speaking (with preparation)	5.64 (0.93)	6.07 (1.53)	N/A	4.94 (1.30)	5.75 (1.06)	N/A
IELTS Listening proficiency	15.13 (3.66)	16.50 (3.08)	N/A	13.06 (3.90)	16.00 (3.32)	N/A
Can-do (productive)	3.09 (0.46)	3.70 (0.30)	N/A	3.25 (0.83)	3.80 (0.79)	N/A
Can-do (receptive)	3.55 (0.58)	4.01 (0.41)	N/A	3.62 (0.89)	4.03 (0.77)	N/A

N/A: English proficiency was assessed before and after the intervention in a separate testing session taking approximately 45 min.

**Table 4:** Planned comparison results for the significant group × time × variable interactions.

Difference Between Intervention Groups per Time-Point for Psychosocial and Cognitive Outcomes							
Contrast	Group	Variable	est	*SE*	*t*	*p*	*d*
control—(past) depression	pretest	AES	0.81	0.33	2.47	<0.05	0.89
control—(past) depression	pretest	BRS	1.27	0.33	3.89	<0.001	1.41
control—(past) depression	pretest	CFQ	1.22	0.33	3.74	<0.001	1.35
control—(past) depression	pretest	Digit span (forward)	0.71	0.33	2.16	<0.05	0.78
control—(past) depression	pretest	GDS	1.19	0.33	3.65	<0.001	1.32
control—(past) depression	pretest	LARSS	0.69	0.33	2.1	<0.05	0.76
control—(past) depression	pretest	loneliness (emotional)	0.88	0.33	2.68	<0.01	0.97
control—(past) depression	pretest	loneliness (social)	1.37	0.33	4.19	<0.001	1.52
control—(past) depression	pretest	MoCA	0.96	0.33	2.95	<0.01	1.07
control—(past) depression	posttest	BRS	0.95	0.33	2.91	<0.01	1.05
control—(past) depression	posttest	CFQ	0.64	0.33	1.97	<0.05	0.71
control—(past) depression	posttest	Digit span (backward)	0.82	0.33	2.5	<0.05	0.9
control—(past) depression	posttest	Digit span (forward)	0.82	0.33	2.52	<0.05	0.91
control—(past) depression	posttest	LARSS	0.89	0.33	2.72	<0.01	0.99
control—(past) depression	posttest	letter-number seq	1.34	0.33	4.09	<0.001	1.48
control—(past) depression	posttest	loneliness (emotional)	0.76	0.33	2.33	<0.05	0.84
control—(past) depression	posttest	loneliness (social)	0.75	0.33	2.3	<0.05	0.83
control—(past) depression	followup	AES	0.83	0.34	2.42	<0.05	0.92
control—(past) depression	followup	BRS	1.06	0.33	3.2	<0.01	1.17
control—(past) depression	followup	LARSS	0.69	0.33	2.09	<0.05	0.77
control—(past) depression	followup	loneliness (emotional)	0.89	0.33	2.69	<0.01	0.98
control—(past) depression	followup	MoCA	0.88	0.34	2.63	<0.01	0.98
control—(past) depression	followup	VF	0.66	0.33	2.01	<0.05	0.74
**Differences over time within groups for psychosocial and cognitive outcomes**							
**Contrast**	**Group**	**Variable**	**est**	***SE***	***t***	***p***	***d***
pretest—posttest	(past) depression	MoCA	−0.83	0.29	−2.83	<0.01	−0.92
pretest—posttest	(past) depression	AES	−0.59	0.3	−2	<0.05	−0.66
pretest—posttest	(past) depression	Loneliness (social)	−0.64	0.29	−2.17	<0.05	−0.7
pretest—followup	(past) depression	CFQ	−0.72	0.3	−2.42	<0.05	−0.8
pretest—followup	(past) depression	VAT-E (paired assoc)	−0.85	0.3	−2.86	<0.01	−0.94
pretest—followup	(past) depression	Loneliness (social)	−0.62	0.3	−2.09	<0.05	−0.69
pretest—posttest	control	Letter-number seq	−1	0.33	−3.02	<0.01	−1.1
pretest—followup	control	VAT-E (paired assoc)	−0.8	0.33	−2.43	<0.05	−0.89
posttest—followup	control	Letter-number seq	1.23	0.34	3.67	<0.001	1.36
**Difference over time between intervention groups for psychosocial and cognitive outcomes**							
**Time (pairwise)**	**Group (pairwise)**	**Variable**	**est**	***SE***	***t***	***p***	***d***
pretest—posttest	control—(past) depression	AES	0.91	0.44	2.05	<0.05	1.01
posttest—followup	control—(past) depression	AES	−0.93	0.46	−2.04	<0.05	−1.03
posttest—followup	control—(past) depression	Letter-number seq	1.12	0.45	2.5	<0.05	1.24
**Difference between groups per time-point for English proficiency outcomes**							
**Contrast**	**Time**	**Variable**	**est**	***SE***	***t***	***p***	***d***
control—(past) depression	pretest	IELTS (listening)	0.7	0.33	2.16	<0.05	0.93
control—(past) depression	pretest	IELTS (speaking w/o prep)	0.72	0.32	2.22	<0.05	0.95
control—(past) depression	pretest	IELTS (speaking with prep)	0.65	0.33	1.98	<0.05	0.86
control—(past) depression	posttest	VF	0.9	0.33	2.75	<0.01	1.2
**Differences over time within group for English proficiency outcomes**							
**Contrast**	**Group**	**Variable**	**est**	***SE***	***t***	***p***	***d***
pretest—posttest	(past) depression	IELTS (listening)	−0.77	0.29	−2.63	<0.01	−1.03
pretest—posttest	(past) depression	IELTS (speaking w/o prep)	−0.8	0.26	−3.12	<0.01	−1.06
pretest—posttest	(past) depression	IELTS (speaking with prep)	−0.68	0.26	−2.61	<0.01	−0.9
pretest—posttest	(past) depression	VF	−0.54	0.26	−2.12	<0.05	−0.72
pretest—posttest	(past) depression	Can-do (productive)	−0.79	0.25	−3.16	<0.01	−1.04
pretest—posttest	(past) depression	Can-do (receptive)	−0.61	0.24	−2.5	<0.05	−0.81
pretest—posttest	control	VF	−1.03	0.29	−3.59	<0.001	−1.36
pretest—posttest	control	Can-do (productive)	−0.9	0.28	−3.28	<0.01	−1.2
pretest—posttest	control	Can-do (receptive)	−0.66	0.28	−2.4	<0.05	−0.88

## Data Availability

The original data presented in the study are openly available in Open Science Framework at [https://osf.io/sa94q, accessed on 11 September 2025].

## Supplementary Materials

The following supporting information can be downloaded at: https://www.mdpi.com/article/10.3390/brainsci15090991/s1, File S1: Model building and results; Figure S2: Flowchart for inclusion in the study; File S3: Complete results including non-significant findings.
