# Supplementary material for: Language Learning as a Non-Pharmacological Intervention in Older Adults with (Past) Depression
Source: Brain Sci. 2025 Sep 15;15(9):991. doi: 10.3390/brainsci15090991 (PMC12468076; doi:10.3390/brainsci15090991)
Supplement: Supplementary file 1 [file brainsci-15-00991-s001.zip › S1 - model building, full results & plots.html]

Intervention analysis CogFlex project (LLD & HC late-life language learners


# Intervention analysis CogFlex project (LLD & HC late-life language learners

#### Jelle Brouwer1, Floor van den Berg1, Remco Knooihuizen1, Hanneke Loerts1, & Merel Keijzer1 1University of Groningen, The Netherlands

#### 28-04-2023

## Description

This Rmd file gives an example of how multiple DVs can be included
into a single model. The method is based on r-scripts from colleagues,
and this blog-post from Ben
Bolker.

**Very short summary of the study** Healthy older adults
and older adults with a history of depression followed an English course
to see if these had an effect on their cognitive functioning and
well-being. Data was collected before the study (pretest), after the
study (posttest), and four months after the course had ended
(followup).

**Please note:** The healthy control group of this study
was also analysed in a different study in this project, which was a
randomized controlled trial (RCT) comparing different types of training
interventions in older adults (lectures, music, and
**language**).

**I’ve made quite a lot of notes in the rmd file but the basic
process goes as follows**

- Select outcome variabes
- Invert outcome variables where necessary so that an increase in
  score always means improvement
- Z-scale and center the variables so that each variable is measured
  on the same scale
- Melt the DF, so that there is one column with outcomes and another
  column indicating which variable is measured
- Run a lmer with a random intercept for participant. Rerun lmer with
  gam and family set to scaled-t
- Interpret results using pairwise comparisons with emmeans

## Legend

| Variable name | Measures |
| --- | --- |
| PP | Participant ID |
| age | age in years |
| group | participant group (healthy control; MCI; LLD) |
| intervention | course (language, lecture, music) |
| sex | female/male |
| nlv\_dart | IQ estimation from Dutch Adult Reading Task (Schmand et al., 1991) |
| cfq totaal | total score on cognitive failures questionnaire (Broadbent et al., 1982) |
| moca\_score | score on montreal cognitive assessment |
| vat\_e\_gepaarde\_associatie, vat\_e\_vrije\_reproductie | subsections of the visual association task-extended (Meyer et al., 2017) |
| symboolsubstitutie, cijferreeksen\_vooruit\_aantal\_goed, cijferreeksen\_achteruit\_aantal\_goed, cijfer\_letter\_reeksen\_aantal\_goed | subtests (digit symbol substitution test (DSST), digit-span forward & backward, letter-number sequencing) of the WAIS-IV |
| tmt\_a\_tijd\_in\_sec | Time to complete TMT-A |
| tmt\_b\_tijd\_in\_sec | Time to complete TMT-B |
| vf\_total | Total number of correctly named words on phonetic verbal fluency task |
| aes\_score\_self | Apathy evaluation scale score (Sheikh & Yesavage, 1986) |
| gds\_15\_score | Geriatric Depression Scale (short form) score (Marin et al., 1991) |
| erq\_score\_cognitive\_reappraisal | Emotion regulation questionnaire (reappraisal) (Gross & John, 2003) |
| erq\_score\_expressive\_suppression | Emotion regulation questionnaire (suppression) (Gross & John, 2003) |
| larss\_score\_totaal | Leuven adaptation of the rumination on sadness scale (Raes et al., 2007) |
| loneliness\_scale\_emotional | Emotional loneliness (de Jong-Gierveld & van Tilburg, 2006) |
| loneliness\_scale\_social | Social Loneliness (de Jong-Gierveld & van Tilburg, 2006) |
| brs\_score | Brief resilience scale (Smith et al., 2008) |
| ppvt\_raw | Peabody picture vocabulary naming test iv (Dunn, 2007) |
| ielts\_ls\_raw | IELTS listening task (IELTS) |
| ielts\_sp\_with\_prep | IELTS speaking task with preparation (IELTS) |
| ielts\_sp\_without\_prep | IELTS speaking task without preparation (IELTS) |
| vf\_eng\_total | Total number of correctly named words on English phonetic verbal fluency task |
| ENG\_CANDO\_PRODUCTIVE | Mean of can-do scales regarding English production skills (1-5) |
| ENG\_CANDO\_RECEPTIVE | Mean of can-do scales regarding English reception skills (1-5) |

## Loading packages

```
library(dplyr) # For more legible code (version 1.1.0)
library(reshape2) # For reshaping the DF (version 1.4.4)
library(FSA) # For descriptives (version 0.9.4)

library(lme4) # For running regression (version 1.1-31)
library(lmerTest) # For forcing lme4 to give p-values (version 3.1-3)
library(performance) # For model checks (version 0.10.2)
library(mgcv) # For running regression as GAM (version 1.8-41)

library(interactions) 
library(ggplot2) # For making the plots a bit more interpretable (version 3.4.1)
library(emmeans) # For planned comparisons between groups (version 1.8.4-1)
library(parameters) # For extracting model parameters (version 0.20.2)
library(interactions) # For plotting results (version 1.1.5)

library(knitr) # Make cool looking tables
library(kableExtra)
options(scipen=999) # Turn off scientific notation
```

# Cognitive & socio-affective model

## Load data & select variables

We’ll start by loading the raw data. Since the raw data contains a
large number of variables (174, to be precise), we’ll make a subset of
the variables that will be used in the analysis. This will make it much
easier to keep an overview.

```
load(file="Y:/staff/let/BALAB/CogFlex/Data/Complete data/intervention_data_complete_30-01-2023.Rdata") # Load data

# select columns
data_wide = data %>% select("PP",starts_with(c(
                                       # Covariates
                                       "age",
                                       "group",
                                       "intervention", 
                                       "time",
                                       "cri", 
                                       "av_exp",
                                       "geslacht",                        
                                       "nlv_dart_score_gecorrigeerd_voor_leeftijd_geslacht",
                                       "gds_15_score_screening",
                                       "scid_5_huidige_stemmingsepisode_screening",
                                       
                                       # Cognition
                                       "cfq_totaal",                                                     
                                       "moca_score", 
                                       "vat_e_g",
                                       "vat_e_v",
                                       "symboolsubs",
                                       "cijferreeksen_vooruit_aantal_goed",
                                       "cijferreeksen_achteruit_aantal_goed",
                                       "cijfer_letter_reeksen_aantal_goed",
                                       "tmt_a",
                                       "tmt_b_tijd",
                                       "totaal_totaal",
                                       
                                       # Socio-affect / well-being
                                       "aes_score_self",
                                       "gds_15_score",
                                       "erq", 
                                       "larss_score_totaal", 
                                       "loneliness_scale_", 
                                       "brs" 
))) %>%  select(-ends_with(c("moca_score_screening", "moca_versie_screening","nodig_screening", "uitgestelde_herkenning", "beroep", "vrije_tijd", "opleiding", "informant")))  %>% filter((group == "HC" | group == "LLD") & intervention == "Taal")

# Make some column names a bit more informative
data_wide = plyr::rename(data_wide, c("geslacht" = "sex",
                                      "nlv_dart_score_gecorrigeerd_voor_leeftijd_geslacht" = "nlv_dart",
                                      "cijferreeksen_vooruit_aantal_goed" = "digit_span_forward",
                                      "cijferreeksen_achteruit_aantal_goed" = "digit_span_backward",
                                      "cijfer_letter_reeksen_aantal_goed" = "letter_number_seq",
                                      "totaal_totaal_aantal_woorden" = "vf_total"))

data_wide = droplevels(data_wide) # Drop unused levels

data_wide = data_wide %>% mutate(group = recode(group, "HC" = "Control", "LLD" = "(past) depression")) # Recode group variable so it is more informative
```

## Descriptives

Let us now look at some descriptives per intervention group. We’ll
start with background variables (e.g., age, sex), and we’ll end with the
raw data per test, group, andm moment in time.

```
# Subset on some cols for descriptives and only select pretest data (because otherwise it will count everything 3 times)
descr = data %>% select("PP",starts_with(c(
                                       # Covariates
                                       "age",
                                       "intervention",
                                       "group", 
                                       "time",
                                       "cri", 
                                       "werksituatie_nu",
                                       "geslacht",                        
                                       "nlv_dart_score_gecorrigeerd_voor_leeftijd_geslacht",
                                       "moca_score_screening",
                                       "tipi",
                                       "av_exp",
                                       "gds_15_score_screening",
                                       "scid",
                                       
                                       # Cognition
                                       "cfq_totaal"))) %>% filter(intervention == "Taal" & time == "pretest" & group != "MCI")
  
descr = droplevels(descr) # Drop unused levels

# Show summary statistics
# Age
Summarize(age~ group, data=descr, digits=2)
```

```
##   group  n  mean  sd min   Q1 median   Q3 max
## 1    HC 15 70.13 3.8  65 67.0     69 74.0  76
## 2   LLD 19 69.74 2.9  66 67.5     69 71.5  75
```

```
# Sex
table(descr$geslacht, descr$group)
```

```
##        
##         HC LLD
##   man    6   4
##   vrouw  9  15
```

```
prop.table(table(descr$geslacht, descr$group),2)
```

```
##        
##                HC       LLD
##   man   0.4000000 0.2105263
##   vrouw 0.6000000 0.7894737
```

```
# Working situation
table(descr$werksituatie_nu, descr$group)
```

```
##                                                             
##                                                              HC LLD
##   2. Part-time betaald werkzaam (minder dan 40 uur per week)  1   1
##   3. Vrijwilligerswerk (onbetaald werkzaam)                   1   1
##   4. Eigen baas                                               1   0
##   8. Mantelzorger                                             1   0
##   9. Gepensioneerd                                           11  17
```

```
prop.table(table(descr$werksituatie_nu, descr$group),2)
```

```
##                                                             
##                                                                      HC
##   2. Part-time betaald werkzaam (minder dan 40 uur per week) 0.06666667
##   3. Vrijwilligerswerk (onbetaald werkzaam)                  0.06666667
##   4. Eigen baas                                              0.06666667
##   8. Mantelzorger                                            0.06666667
##   9. Gepensioneerd                                           0.73333333
##                                                             
##                                                                     LLD
##   2. Part-time betaald werkzaam (minder dan 40 uur per week) 0.05263158
##   3. Vrijwilligerswerk (onbetaald werkzaam)                  0.05263158
##   4. Eigen baas                                              0.00000000
##   8. Mantelzorger                                            0.00000000
##   9. Gepensioneerd                                           0.89473684
```

```
# CRI
Summarize(cri~ group, data=descr, digits=2)
```

```
##   group  n   mean    sd min    Q1 median    Q3 max
## 1    HC 15 144.67 19.70 113 131.5    147 156.0 183
## 2   LLD 19 131.21 13.54 111 121.0    131 137.5 160
```

```
Summarize(cri_beroep~ group, data=descr, digits=2)
```

```
##   group  n   mean    sd min  Q1 median  Q3 max
## 1    HC 15 130.47 20.47  94 117    133 139 169
## 2   LLD 19 104.63 17.57  82  90    101 116 143
```

```
Summarize(cri_opleiding~ group, data=descr, digits=2)
```

```
##   group  n   mean    sd min  Q1 median    Q3 max
## 1    HC 15 133.07 16.00 112 121    135 146.5 158
## 2   LLD 19 131.21 12.15 112 123    125 143.5 151
```

```
Summarize(cri_vrije_tijd~ group, data=descr, digits=2)
```

```
##   group  n   mean    sd min    Q1 median    Q3 max
## 1    HC 15 137.67 21.35  94 127.0    139 146.5 181
## 2   LLD 19 133.37 18.29 107 120.5    130 146.0 174
```

```
# IQ
Summarize(nlv_dart_score_gecorrigeerd_voor_leeftijd_geslacht~ group, data=descr, digits=2)
```

```
##   group  n  mean   sd   min    Q1 median     Q3    max
## 1    HC 15 92.46 8.52 78.49 86.82  92.32  95.71 106.20
## 2   LLD 19 93.95 8.21 77.89 90.78  94.07 100.12 105.36
```

```
# TIPI
Summarize(tipi_score_conscientieusheid~ group, data=descr, digits=2)
```

```
##   group  n mean   sd min   Q1 median   Q3 max
## 1    HC 15 5.10 0.95 3.0 4.75      5 5.75 6.5
## 2   LLD 19 4.76 1.26 2.5 4.00      5 5.50 7.0
```

```
Summarize(tipi_score_extravert~ group, data=descr, digits=2)
```

```
##   group  n mean   sd min  Q1 median Q3 max
## 1    HC 15 4.93 0.62   4 4.5      5  5 6.5
## 2   LLD 19 4.16 1.42   2 3.0      4  5 7.0
```

```
Summarize(tipi_score_neuroticisme~ group, data=descr, digits=2)
```

```
##   group  n mean   sd min  Q1 median   Q3 max
## 1    HC 15 5.80 0.94 3.5 5.5    6.0 6.50 7.0
## 2   LLD 19 4.45 1.54 2.5 3.0    4.5 5.75 6.5
```

```
Summarize(tipi_score_openheid~ group, data=descr, digits=2)
```

```
##   group  n mean   sd min  Q1 median  Q3 max
## 1    HC 15 5.33 1.50 3.0 4.5    5.5 6.5   7
## 2   LLD 19 5.55 1.22 3.5 4.5    6.0 6.5   7
```

```
Summarize(tipi_score_vriendelijkheid~ group, data=descr, digits=2)
```

```
##   group  n mean   sd min   Q1 median   Q3 max
## 1    HC 15 5.63 0.77   4 5.25    5.5 6.25 6.5
## 2   LLD 19 5.79 0.69   4 5.50    6.0 6.00 7.0
```

```
# Moca at screening
Summarize(moca_score_screening~ group, data=descr, digits=2)
```

```
##   group  n nvalid  mean   sd min Q1 median    Q3 max
## 1    HC 15     15 26.87 2.17  24 25     28 28.00  30
## 2   LLD 19     18 25.06 2.36  21 24     25 25.75  30
```

```
Summarize(cfq_totaal~ group, data=descr, digits=2)
```

```
##   group  n  mean    sd min Q1 median   Q3 max
## 1    HC 15 31.40 11.68  10 27     29 37.5  54
## 2   LLD 19 46.11 13.43  29 35     43 57.0  70
```

```
# Course exposure
Summarize(av_exp~ group, data=descr, digits=2)
```

```
##   group  n nvalid   mean     sd min     Q1 median    Q3 max
## 1    HC 15     13 344.38 257.63  95 178.00  222.0 505.0 977
## 2   LLD 19     18 311.17 119.80   9 252.75  326.5 377.5 530
```

```
# English level at baseline
Summarize(ielts_sp_with_prep~ group, data=data %>% filter(group != "MCI"& time == "pretest"), digits=2)
```

```
##   group  n nvalid mean   sd min Q1 median   Q3 max
## 1    HC 43     14 5.64 0.93   4  5      6 6.00   7
## 2   LLD 19     18 4.94 1.30   2  4      5 5.75   7
```

```
# Course evaluation (needs to be subsetted again because this was conducted at posttest)
course = data %>% select(starts_with(c("group", "time", "EVAL", "intervention"))) %>%  filter((group == "HC" | group == "LLD") & intervention == "Taal"  & time == "posttest")
```

```
## Adding missing grouping variables: `PP`
```

```
Summarize(EVAL_SUM_SOCIAL~group, data=course, digits=2)
```

```
##   group  n nvalid mean   sd min Q1 median   Q3 max
## 1    HC 15     14 4.37 0.55 3.2  4    4.3 4.95   5
## 2   LLD 19     18 4.23 0.99 1.0  4    4.5 4.95   5
```

```
Summarize(EVAL_SUM_INSTRUCTOR~group, data=course, digits=2)
```

```
##   group  n nvalid mean   sd  min   Q1 median Q3 max
## 1    HC 15     14 4.76 0.43 3.57 4.64   5.00  5   5
## 2   LLD 19     18 4.49 0.96 1.00 4.46   4.86  5   5
```

```
# Check if variables differ at baseline/screening
fisher.test(table(descr$geslacht, descr$group))
```

```
## 
##  Fisher's Exact Test for Count Data
## 
## data:  table(descr$geslacht, descr$group)
## p-value = 0.2764
## alternative hypothesis: true odds ratio is not equal to 1
## 95 percent confidence interval:
##   0.4362475 15.2612039
## sample estimates:
## odds ratio 
##   2.430909
```

```
fisher.test(table(descr$werksituatie_nu, descr$group))
```

```
## 
##  Fisher's Exact Test for Count Data
## 
## data:  table(descr$werksituatie_nu, descr$group)
## p-value = 0.7882
## alternative hypothesis: two.sided
```

```
wilcox.test(descr$age~ descr$group, paired=F)
```

```
## Warning in wilcox.test.default(x = DATA[[1L]], y = DATA[[2L]], ...): cannot
## compute exact p-value with ties
```

```
## 
##  Wilcoxon rank sum test with continuity correction
## 
## data:  descr$age by descr$group
## W = 149, p-value = 0.8342
## alternative hypothesis: true location shift is not equal to 0
```

```
wilcox.test(descr$moca_score_screening~ descr$group)
```

```
## Warning in wilcox.test.default(x = DATA[[1L]], y = DATA[[2L]], ...): cannot
## compute exact p-value with ties
```

```
## 
##  Wilcoxon rank sum test with continuity correction
## 
## data:  descr$moca_score_screening by descr$group
## W = 189.5, p-value = 0.04762
## alternative hypothesis: true location shift is not equal to 0
```

```
wilcox.test(descr$cri~ descr$group)
```

```
## Warning in wilcox.test.default(x = DATA[[1L]], y = DATA[[2L]], ...): cannot
## compute exact p-value with ties
```

```
## 
##  Wilcoxon rank sum test with continuity correction
## 
## data:  descr$cri by descr$group
## W = 204, p-value = 0.0342
## alternative hypothesis: true location shift is not equal to 0
```

```
wilcox.test(descr$nlv_dart_score_gecorrigeerd_voor_leeftijd_geslacht~ descr$group)
```

```
## 
##  Wilcoxon rank sum exact test
## 
## data:  descr$nlv_dart_score_gecorrigeerd_voor_leeftijd_geslacht by descr$group
## W = 128, p-value = 0.6316
## alternative hypothesis: true location shift is not equal to 0
```

```
wilcox.test(descr$av_exp~ descr$group)
```

```
## 
##  Wilcoxon rank sum exact test
## 
## data:  descr$av_exp by descr$group
## W = 98, p-value = 0.4646
## alternative hypothesis: true location shift is not equal to 0
```

```
wilcox.test(course$EVAL_SUM_SOCIAL~ course$group)
```

```
## Warning in wilcox.test.default(x = DATA[[1L]], y = DATA[[2L]], ...): cannot
## compute exact p-value with ties
```

```
## 
##  Wilcoxon rank sum test with continuity correction
## 
## data:  course$EVAL_SUM_SOCIAL by course$group
## W = 125.5, p-value = 1
## alternative hypothesis: true location shift is not equal to 0
```

```
wilcox.test(course$EVAL_SUM_INSTRUCTOR~ course$group)
```

```
## Warning in wilcox.test.default(x = DATA[[1L]], y = DATA[[2L]], ...): cannot
## compute exact p-value with ties
```

```
## 
##  Wilcoxon rank sum test with continuity correction
## 
## data:  course$EVAL_SUM_INSTRUCTOR by course$group
## W = 154, p-value = 0.2627
## alternative hypothesis: true location shift is not equal to 0
```

```
temp = data %>% filter(group != "MCI" & time == "pretest")
wilcox.test(temp$ielts_sp_with_prep~ temp$group)
```

```
## Warning in wilcox.test.default(x = DATA[[1L]], y = DATA[[2L]], ...): cannot
## compute exact p-value with ties
```

```
## 
##  Wilcoxon rank sum test with continuity correction
## 
## data:  temp$ielts_sp_with_prep by temp$group
## W = 169, p-value = 0.09534
## alternative hypothesis: true location shift is not equal to 0
```

```
cando_pre = data %>% select(starts_with(c("group", "time", "ENG_CANDO", "intervention"))) %>%  filter((group == "HC" | group == "LLD") & intervention == "Taal"  & time == "pretest")
```

```
## Adding missing grouping variables: `PP`
```

```
wilcox.test(cando_pre$ENG_CANDO ~ cando_pre$group)
```

```
## Warning in wilcox.test.default(x = DATA[[1L]], y = DATA[[2L]], ...): cannot
## compute exact p-value with ties
```

```
## 
##  Wilcoxon rank sum test with continuity correction
## 
## data:  cando_pre$ENG_CANDO by cando_pre$group
## W = 104.5, p-value = 0.2776
## alternative hypothesis: true location shift is not equal to 0
```

```
cando_post = data %>% select(starts_with(c("group", "time", "ENG_CANDO", "intervention"))) %>%  filter((group == "HC" | group == "LLD") & intervention == "Taal"  & time == "posttest")
```

```
## Adding missing grouping variables: `PP`
```

```
wilcox.test(cando_post$ENG_CANDO ~ cando_pre$group)
```

```
## Warning in wilcox.test.default(x = DATA[[1L]], y = DATA[[2L]], ...): cannot
## compute exact p-value with ties
```

```
## 
##  Wilcoxon rank sum test with continuity correction
## 
## data:  cando_post$ENG_CANDO by cando_pre$group
## W = 96.5, p-value = 0.1142
## alternative hypothesis: true location shift is not equal to 0
```

```
# Raw data averages
lapply(data_wide[12:30], function(x) Summarize(x ~group*time, data=data_wide, digits=2))
```

```
## $cfq_totaal
##               group     time  n  mean    sd min    Q1 median    Q3 max
## 1           Control  pretest 15 31.40 11.68  10 27.00   29.0 37.50  54
## 2 (past) depression  pretest 19 46.11 13.43  29 35.00   43.0 57.00  70
## 3           Control posttest 15 32.20  8.13  19 26.50   31.0 39.00  42
## 4 (past) depression posttest 19 39.89  8.37  28 34.50   40.0 43.50  60
## 5           Control followup 15 30.33 10.41   9 24.50   34.0 37.50  46
## 6 (past) depression followup 18 37.56 12.21  22 28.75   35.5 41.25  64
## 
## $moca_score
##               group     time  n nvalid  mean   sd min    Q1 median Q3 max
## 1           Control  pretest 15     15 26.93 1.87  24 26.00     28 28  30
## 2 (past) depression  pretest 19     19 24.95 2.46  21 23.50     25 27  30
## 3           Control posttest 15     15 27.07 2.25  23 25.50     27 29  30
## 4 (past) depression posttest 19     19 26.63 1.67  24 25.00     26 28  29
## 5           Control followup 15     14 27.71 1.14  25 27.25     28 28  29
## 6 (past) depression followup 18     18 25.83 1.69  22 25.00     26 27  29
## 
## $vat_e_gepaarde_associatie
##               group     time  n  mean   sd min    Q1 median   Q3 max
## 1           Control  pretest 15 14.33 4.82   5 12.00   15.0 17.5  22
## 2 (past) depression  pretest 19 14.58 6.21   4  9.00   16.0 19.0  24
## 3           Control posttest 15 17.33 6.26   3 14.50   18.0 22.5  24
## 4 (past) depression posttest 19 17.63 6.95   2 14.50   20.0 23.0  24
## 5           Control followup 15 19.07 5.81   4 16.00   21.0 23.5  24
## 6 (past) depression followup 18 19.72 4.96   7 18.25   21.5 23.0  24
## 
## $vat_e_vrije_reproductie
##               group     time  n  mean    sd min   Q1 median   Q3 max
## 1           Control  pretest 15 24.93  7.52  10 20.5     26 30.5  35
## 2 (past) depression  pretest 19 20.89  8.03   8 16.5     18 25.0  36
## 3           Control posttest 15 28.53  9.36  10 23.0     28 36.5  42
## 4 (past) depression posttest 19 25.42  7.81  10 21.0     28 30.0  44
## 5           Control followup 15 29.67 10.33  12 23.0     30 36.5  48
## 6 (past) depression followup 18 25.61  8.68  10 21.0     25 29.5  44
## 
## $symboolsubstitutie
##               group     time  n nvalid  mean    sd min    Q1 median    Q3 max
## 1           Control  pretest 15     12 60.67  7.52  47 56.75   60.5 66.25  72
## 2 (past) depression  pretest 19     18 54.06 14.98  36 43.25   50.5 63.75  93
## 3           Control posttest 15     13 63.38  9.12  50 57.00   63.0 68.00  81
## 4 (past) depression posttest 19     19 57.68 17.58  39 46.00   53.0 63.00 113
## 5           Control followup 15     15 64.67  9.42  47 60.00   64.0 69.50  86
## 6 (past) depression followup 18     15 58.33 13.96  37 51.50   58.0 63.50  98
## 
## $digit_span_forward
##               group     time  n nvalid mean   sd min   Q1 median    Q3 max
## 1           Control  pretest 15     15 7.20 2.18   2 6.00      8  8.50  10
## 2 (past) depression  pretest 19     19 5.74 1.41   4 4.50      6  6.50   8
## 3           Control posttest 15     15 7.60 2.69   3 5.50      7 10.00  12
## 4 (past) depression posttest 19     19 5.89 1.73   2 5.00      6  7.00   9
## 5           Control followup 15     14 6.57 2.03   3 6.00      6  7.75  11
## 6 (past) depression followup 18     18 5.83 2.04   2 4.25      6  7.00   9
## 
## $digit_span_backward
##               group     time  n nvalid mean   sd min   Q1 median   Q3 max
## 1           Control  pretest 15     15 7.60 1.92   5 6.50    7.0  8.0  12
## 2 (past) depression  pretest 19     19 6.37 1.38   4 5.50    6.0  8.0   8
## 3           Control posttest 15     15 8.27 2.31   4 7.00    8.0 10.0  12
## 4 (past) depression posttest 19     19 6.68 1.63   5 5.50    6.0  7.5  10
## 5           Control followup 15     14 7.43 1.79   5 6.00    7.5  9.0  10
## 6 (past) depression followup 18     18 6.61 2.20   2 6.25    7.0  8.0  10
## 
## $letter_number_seq
##               group     time  n nvalid  mean   sd min    Q1 median   Q3 max
## 1           Control  pretest 15     15 10.93 2.87   7  9.00   11.0 11.5  17
## 2 (past) depression  pretest 19     19  9.47 1.84   5  8.00   10.0 11.0  13
## 3           Control posttest 15     15 13.53 3.56   6 12.00   14.0 15.0  21
## 4 (past) depression posttest 19     19 10.00 2.00   6  8.50   11.0 11.5  12
## 5           Control followup 15     14 10.36 1.86   8  8.25   10.5 12.0  13
## 6 (past) depression followup 18     18  9.72 1.71   7  9.00   10.0 11.0  13
## 
## $tmt_a_tijd_in_sec
##               group     time  n  mean    sd min   Q1 median   Q3 max
## 1           Control  pretest 15 38.73  8.22  23 32.5   39.0 44.0  52
## 2 (past) depression  pretest 19 44.79 12.49  30 35.0   42.0 52.0  68
## 3           Control posttest 15 36.93  6.49  23 35.0   40.0 41.5  44
## 4 (past) depression posttest 19 41.84 12.02  26 32.0   37.0 50.5  66
## 5           Control followup 15 37.40  7.83  22 33.0   35.0 42.0  52
## 6 (past) depression followup 18 41.39 12.22  28 32.5   38.5 44.5  70
## 
## $tmt_b_tijd_in_sec
##               group     time  n  mean    sd min   Q1 median    Q3 max
## 1           Control  pretest 15 78.53 19.46  45 66.5     76 91.00 119
## 2 (past) depression  pretest 19 87.26 24.49  58 74.0     82 97.00 156
## 3           Control posttest 15 79.93 26.33  45 62.0     76 91.00 156
## 4 (past) depression posttest 19 90.95 35.68  53 72.0     85 99.00 224
## 5           Control followup 15 74.53 19.71  51 58.5     66 90.00 111
## 6 (past) depression followup 18 78.50 16.22  49 70.5     78 91.75 106
## 
## $vf_total
##               group     time  n  mean    sd min    Q1 median   Q3 max
## 1           Control  pretest 15 46.53  9.66  28 40.50     47 53.5  64
## 2 (past) depression  pretest 19 44.58 10.30  23 39.50     48 51.0  57
## 3           Control posttest 15 47.00  9.65  25 42.00     46 52.5  66
## 4 (past) depression posttest 19 44.05 11.79  24 37.00     43 50.5  71
## 5           Control followup 15 52.40 12.00  33 42.50     51 61.0  74
## 6 (past) depression followup 18 45.56  9.92  27 39.25     47 52.5  63
## 
## $aes_score_self
##               group     time  n nvalid mean   sd min   Q1 median   Q3 max
## 1           Control  pretest 15     15 26.2 3.86  21 22.5   27.0 29.0  34
## 2 (past) depression  pretest 19     19 31.0 6.77  22 26.0   31.0 34.0  45
## 3           Control posttest 15     15 28.2 5.39  21 25.0   27.0 31.0  40
## 4 (past) depression posttest 19     18 27.5 5.59  20 23.0   27.5 30.0  43
## 5           Control followup 15     15 25.0 4.90  18 21.5   25.0 27.0  37
## 6 (past) depression followup 18     15 30.0 7.25  19 25.5   29.0 34.5  42
## 
## $gds_15_score
##               group     time  n mean   sd min   Q1 median   Q3 max percZero
## 1           Control  pretest 15 0.40 1.30   0 0.00      0 0.00   5    86.67
## 2 (past) depression  pretest 19 4.21 3.55   0 1.50      3 6.50  11    15.79
## 3           Control posttest 15 1.13 2.56   0 0.00      0 1.00  10    60.00
## 4 (past) depression posttest 19 3.00 3.46   0 0.50      1 5.00  11    26.32
## 5           Control followup 15 1.07 2.81   0 0.00      0 1.00  11    66.67
## 6 (past) depression followup 18 3.06 3.24   0 0.25      2 4.75  10    27.78
## 
## $erq_score_cognitive_reappraisal
##               group     time  n  mean   sd min    Q1 median    Q3 max
## 1           Control  pretest 15 28.27 7.27   9 26.00     29 31.00  42
## 2 (past) depression  pretest 19 24.95 5.03  16 21.50     25 27.50  36
## 3           Control posttest 15 26.53 8.87   6 23.00     29 32.00  38
## 4 (past) depression posttest 19 25.84 5.89  12 24.00     26 28.00  37
## 5           Control followup 15 24.33 8.57   6 22.00     26 29.50  35
## 6 (past) depression followup 18 23.28 7.16   7 18.75     25 26.75  34
## 
## $erq_score_expressive_suppression
##               group     time  n  mean   sd min    Q1 median   Q3 max
## 1           Control  pretest 15 13.00 4.11   6 10.50     12 15.5  21
## 2 (past) depression  pretest 19 12.47 5.33   3  9.00     14 15.0  21
## 3           Control posttest 15 13.47 4.16   6 11.00     13 16.0  22
## 4 (past) depression posttest 19 12.00 5.12   4  7.50     13 14.5  23
## 5           Control followup 15 13.67 6.85   4  7.00     17 19.0  22
## 6 (past) depression followup 18 12.61 4.17   4 11.25     13 14.0  21
## 
## $larss_score_totaal
##               group     time  n  mean    sd min   Q1 median   Q3 max
## 1           Control  pretest 15 32.67 14.52  21 21.0   25.0 43.5  70
## 2 (past) depression  pretest 19 43.58 14.49  23 32.5   44.0 49.5  75
## 3           Control posttest 15 28.27 15.41  21 21.0   22.0 28.0  82
## 4 (past) depression posttest 19 42.47 14.01  23 32.5   40.0 54.5  65
## 5           Control followup 15 33.33 16.47  21 21.0   25.0 42.5  80
## 6 (past) depression followup 18 44.06 16.46  22 32.0   42.5 58.0  78
## 
## $loneliness_scale_score_emotional
##               group     time  n mean   sd min   Q1 median   Q3 max percZero
## 1           Control  pretest 15 0.20 0.77   0 0.00      0 0.00   3    93.33
## 2 (past) depression  pretest 19 1.11 1.15   0 0.00      1 2.00   3    42.11
## 3           Control posttest 15 0.27 0.80   0 0.00      0 0.00   3    86.67
## 4 (past) depression posttest 19 1.05 1.08   0 0.00      1 2.00   3    42.11
## 5           Control followup 15 0.27 0.80   0 0.00      0 0.00   3    86.67
## 6 (past) depression followup 18 1.17 1.04   0 0.25      1 1.75   3    27.78
## 
## $loneliness_scale_score_social
##               group     time  n mean   sd min  Q1 median   Q3 max percZero
## 1           Control  pretest 15 0.27 0.59   0 0.0    0.0 0.00   2    80.00
## 2 (past) depression  pretest 19 1.89 1.33   0 0.5    3.0 3.00   3    26.32
## 3           Control posttest 15 0.27 0.80   0 0.0    0.0 0.00   3    86.67
## 4 (past) depression posttest 19 1.16 0.90   0 0.5    1.0 2.00   3    26.32
## 5           Control followup 15 0.47 1.06   0 0.0    0.0 0.00   3    80.00
## 6 (past) depression followup 18 1.17 1.34   0 0.0    0.5 2.75   3    50.00
## 
## $brs_score
##               group     time  n mean   sd  min   Q1 median   Q3  max
## 1           Control  pretest 15 3.91 0.43 3.17 3.66   3.83 4.08 5.00
## 2 (past) depression  pretest 19 3.08 0.55 2.17 2.50   3.33 3.50 4.00
## 3           Control posttest 15 3.69 0.69 2.00 3.58   3.83 4.00 5.00
## 4 (past) depression posttest 19 3.07 0.51 2.33 2.75   2.83 3.50 3.83
## 5           Control followup 15 3.79 0.62 2.50 3.67   3.67 4.08 5.00
## 6 (past) depression followup 18 3.11 0.56 2.17 2.83   3.08 3.50 4.00
```

## Preparing the dataframe for analysis

For some of the DVs we have, a higher score is negative (e.g., higher
CFQ score means more cognitive failures), for others higher is positive
(e.g., a higher BRS score means someone is more resilient). To make
interpretability easier, we reverse some variables, such that a higher
score always represents a positive score.

After this we will melt the dataframe. In essence, this means putting
the scores (value) for each outcome variable into a single column. A
second column (variable) is added to indicate which task/questionnaire
the score represents. At the end of this block you can see an
illustration of what this looks like.

```
# Reverse where necessary
data_wide$cfq_totaal <- data_wide$cfq_totaal *-1
data_wide$tmt_a_tijd_in_sec <- data_wide$tmt_a_tijd_in_sec *-1
data_wide$tmt_b_tijd_in_sec <- data_wide$tmt_b_tijd_in_sec *-1
data_wide$aes_score_self <- data_wide$aes_score_self *-1
data_wide$gds_15_score <- data_wide$gds_15_score *-1
data_wide$erq_score_expressive_suppression <- data_wide$erq_score_expressive_suppression *-1
data_wide$larss_score_totaal <- data_wide$larss_score_totaal *-1
data_wide$loneliness_scale_score_emotional <- data_wide$loneliness_scale_score_emotional *-1
data_wide$loneliness_scale_score_social <- data_wide$loneliness_scale_score_social *-1

# Z-scale all numerical variables
var_list <- colnames(data_wide[12:30])
data_wide[var_list] <- lapply(data_wide[var_list], function(x) scale(x, center=T))

# Melt dataframe (i.e.,: all DVs are put in one column called Value; another column is added that indicates which test is represented by the Value column)
data_melt <- reshape2::melt(data_wide, id.vars=c("PP", "group","age", "time", "intervention", "cri", "av_exp", "sex", "nlv_dart", "scid_5_huidige_stemmingsepisode_screening", "gds_15_score_screening"))
```

```
## Warning: attributes are not identical across measure variables; they will be
## dropped
```

```
head(data_melt %>% select(starts_with(c("value", "variable", "time", "PP", "intervention")))) # Show first couple of rows to illustrate
```

```
##        value   variable    time   PP intervention
## 1  2.1359441 cfq_totaal pretest 1002         Taal
## 2  0.1506954 cfq_totaal pretest 1004         Taal
## 3  0.8951637 cfq_totaal pretest 1008         Taal
## 4 -0.4283354 cfq_totaal pretest 1009         Taal
## 5  0.7297263 cfq_totaal pretest 1011         Taal
## 6  0.6470076 cfq_totaal pretest 1013         Taal
```

## Run analyses and do model checks

Now that we’ve pre-processed the dataset, such that only one model
needs to be built to predict all the cognitive and socio-affective DVs,
we can proceed with building the actual model. We include age, sex, IQ,
and cognitive reserve (an index that includes education level) to the
model as covariates. Additionally, a random intercept is added per
participant.

```
# Run model with all the covariates
m_complete <- lmer(value ~ variable*time*group + age + cri + sex + nlv_dart + (1 | PP), data=data_melt) 

# Run model checks
check_model(m_complete) # Everything seems to be OK; VIF values seem very high but that's caused by interactions
```

```
## Variable `Component` is not in your data frame :/
```

## Results: comparing change within and between groups

we’ve now built our model with cognitive and socio-affective
variables. Next, let’s have a look at the estimates. The
within\_group\_comp DF is used to see if one group improves over time.
Please note that the within\_group\_comp DF looks at ALL comparisons. This
is unnecessary. For instance, we’re not interested in if the GDS score
at follow-up for the music group differs significantly from the pre-test
score for the lecture group, as this comparisons in itself isn’t
interesting (i.e., we need to look at changes over time per group, not
just the raw scores). Therefore, we’ll first use emmeans to run all
comparisons, and then we’ll filter them in DF temp, so that we are left
with the comparisons within each group.

The between group comparisons are made in a different DF. This DF
shows us if a change in a score on a variable over time differs
significantly between groups. Please note that we do not adjust our
p-values, since all comparisons have been planned beforehand. Running
emmeans with an adjustment (e.g., bonferroni) would lead to p-values
that are overly conservative, since it would correct for multiple
comparisons that are made in the code that will not be looked at for the
actual results.

```
est <- emmeans(m_complete, ~time * group | variable, adjust = "none")  # calculate coefficient per task & group
```

```
## Registered S3 methods overwritten by 'broom':
##   method            from  
##   tidy.glht         jtools
##   tidy.summary.glht jtools
```

```
# See if group X has change on score Y over time
within_group <- (pairs(est, simple = "time", adjust = "none"))
eff_within <- as.data.frame(eff_size(within_group, sigma = sigma(m_complete),
    df.residual(m_complete), method = "identity"))  # calculate Cohen's D
within_group = as.data.frame(within_group)
within_group = cbind(within_group, eff_within[4])

# See if group X has change on score Y at a specific point
# in time
over_time <- (pairs(est, simple = "group", adjust = "none"))
eff_over_time <- as.data.frame(eff_size(over_time, sigma = sigma(m_complete),
    df.residual(m_complete), method = "identity"))  # calculate Cohen's D
over_time = as.data.frame(over_time)
over_time = cbind(over_time, eff_over_time[4])

# Compare & contrast time effects by group
between_groups <- (pairs(est, interaction = "pairwise", adjust = "none"))
eff_between_groups <- as.data.frame(eff_size(between_groups,
    sigma = sigma(m_complete), df.residual(m_complete), method = "identity"))  # calculate Cohen's D
between_groups = as.data.frame(between_groups)
between_groups = cbind(between_groups, eff_between_groups[3])

# Show significant differences between group at respective
# timepoints
over_time %>%
    filter(p.value < 0.05) %>%
    mutate_at(4:7, round, 2) %>%
    mutate_at(8, round, 4) %>%
    mutate_at(9, round, 2) %>%
    kbl() %>%
    kable_styling()
```

| contrast | time | variable | estimate | SE | df | t.ratio | p.value | effect.size |
| --- | --- | --- | --- | --- | --- | --- | --- | --- |
| Control - (past) depression | pretest | cfq\_totaal | 1.22 | 0.33 | 1186.10 | 3.74 | 0.0002 | 1.35 |
| Control - (past) depression | posttest | cfq\_totaal | 0.64 | 0.33 | 1185.69 | 1.97 | 0.0495 | 0.71 |
| Control - (past) depression | pretest | moca\_score | 0.96 | 0.33 | 1186.10 | 2.95 | 0.0032 | 1.07 |
| Control - (past) depression | followup | moca\_score | 0.88 | 0.34 | 1237.97 | 2.63 | 0.0087 | 0.98 |
| Control - (past) depression | pretest | digit\_span\_forward | 0.71 | 0.33 | 1186.10 | 2.16 | 0.0306 | 0.78 |
| Control - (past) depression | posttest | digit\_span\_forward | 0.82 | 0.33 | 1185.69 | 2.52 | 0.0120 | 0.91 |
| Control - (past) depression | posttest | digit\_span\_backward | 0.82 | 0.33 | 1185.69 | 2.50 | 0.0125 | 0.90 |
| Control - (past) depression | posttest | letter\_number\_seq | 1.34 | 0.33 | 1185.69 | 4.09 | 0.0000 | 1.48 |
| Control - (past) depression | followup | vf\_total | 0.66 | 0.33 | 1206.47 | 2.01 | 0.0445 | 0.74 |
| Control - (past) depression | pretest | aes\_score\_self | 0.81 | 0.33 | 1186.10 | 2.47 | 0.0138 | 0.89 |
| Control - (past) depression | followup | aes\_score\_self | 0.83 | 0.34 | 1277.28 | 2.42 | 0.0158 | 0.92 |
| Control - (past) depression | pretest | gds\_15\_score | 1.19 | 0.33 | 1186.10 | 3.65 | 0.0003 | 1.32 |
| Control - (past) depression | pretest | larss\_score\_totaal | 0.69 | 0.33 | 1186.10 | 2.10 | 0.0359 | 0.76 |
| Control - (past) depression | posttest | larss\_score\_totaal | 0.89 | 0.33 | 1185.69 | 2.72 | 0.0065 | 0.99 |
| Control - (past) depression | followup | larss\_score\_totaal | 0.69 | 0.33 | 1206.47 | 2.09 | 0.0364 | 0.77 |
| Control - (past) depression | pretest | loneliness\_scale\_score\_emotional | 0.88 | 0.33 | 1186.10 | 2.68 | 0.0074 | 0.97 |
| Control - (past) depression | posttest | loneliness\_scale\_score\_emotional | 0.76 | 0.33 | 1185.69 | 2.33 | 0.0200 | 0.84 |
| Control - (past) depression | followup | loneliness\_scale\_score\_emotional | 0.89 | 0.33 | 1206.47 | 2.69 | 0.0072 | 0.98 |
| Control - (past) depression | pretest | loneliness\_scale\_score\_social | 1.37 | 0.33 | 1186.10 | 4.19 | 0.0000 | 1.52 |
| Control - (past) depression | posttest | loneliness\_scale\_score\_social | 0.75 | 0.33 | 1185.69 | 2.30 | 0.0215 | 0.83 |
| Control - (past) depression | pretest | brs\_score | 1.27 | 0.33 | 1186.10 | 3.89 | 0.0001 | 1.41 |
| Control - (past) depression | posttest | brs\_score | 0.95 | 0.33 | 1185.69 | 2.91 | 0.0037 | 1.05 |
| Control - (past) depression | followup | brs\_score | 1.06 | 0.33 | 1206.47 | 3.20 | 0.0014 | 1.17 |

```
# show significant changes over time
within_group %>%
    filter(p.value < 0.05) %>%
    mutate_at(4:7, round, 2) %>%
    mutate_at(8, round, 4) %>%
    mutate_at(9, round, 2) %>%
    kbl() %>%
    kable_styling()
```

| contrast | group | variable | estimate | SE | df | t.ratio | p.value | effect.size |
| --- | --- | --- | --- | --- | --- | --- | --- | --- |
| pretest - followup | (past) depression | cfq\_totaal | -0.72 | 0.30 | 1759.98 | -2.42 | 0.0157 | -0.80 |
| pretest - posttest | (past) depression | moca\_score | -0.83 | 0.29 | 1757.59 | -2.83 | 0.0047 | -0.92 |
| pretest - followup | Control | vat\_e\_gepaarde\_associatie | -0.80 | 0.33 | 1759.17 | -2.43 | 0.0152 | -0.89 |
| pretest - followup | (past) depression | vat\_e\_gepaarde\_associatie | -0.85 | 0.30 | 1759.98 | -2.86 | 0.0043 | -0.94 |
| pretest - posttest | Control | letter\_number\_seq | -1.00 | 0.33 | 1757.11 | -3.02 | 0.0025 | -1.10 |
| posttest - followup | Control | letter\_number\_seq | 1.23 | 0.34 | 1756.77 | 3.67 | 0.0003 | 1.36 |
| pretest - posttest | (past) depression | aes\_score\_self | -0.59 | 0.30 | 1757.76 | -2.00 | 0.0459 | -0.66 |
| pretest - posttest | (past) depression | loneliness\_scale\_score\_social | -0.64 | 0.29 | 1757.59 | -2.17 | 0.0303 | -0.70 |
| pretest - followup | (past) depression | loneliness\_scale\_score\_social | -0.62 | 0.30 | 1759.98 | -2.09 | 0.0367 | -0.69 |

```
# Show at which points a change over time differed between
# groups
between_groups %>%
    filter(p.value < 0.05) %>%
    mutate_at(4:7, round, 2) %>%
    mutate_at(8, round, 4) %>%
    mutate_at(9, round, 2) %>%
    kbl() %>%
    kable_styling()
```

| time\_pairwise | group\_pairwise | variable | estimate | SE | df | t.ratio | p.value | effect.size |
| --- | --- | --- | --- | --- | --- | --- | --- | --- |
| posttest - followup | Control - (past) depression | letter\_number\_seq | 1.12 | 0.45 | 1756.40 | 2.50 | 0.0125 | 1.24 |
| pretest - posttest | Control - (past) depression | aes\_score\_self | 0.91 | 0.44 | 1756.07 | 2.05 | 0.0408 | 1.01 |
| posttest - followup | Control - (past) depression | aes\_score\_self | -0.93 | 0.46 | 1756.56 | -2.04 | 0.0410 | -1.03 |

## Plotting results for cognition & socio-affect

The emmeans results have shown us if there are changes over time
within each group, and if the magnitude of these changes over time
differs between interventions. To make this more interpretable, we will
also plot the results

```
# Plot everything!!!
cat_plot(m_complete, pred=time, modx=group, mod2=variable, dodge.width = .5, errorbar.width = .33, line.thickness = .85, mod2.labels = c("CFQ", "MoCA", "VAT-E (paired association)", "VAT-E (free recall)", "DSST", "digit span (forward)", "digit span (backward)", "letter-number sequencing", "TMT A (time in sec)", "TMT B (time in sec)", "VF (total correct)", "AES", "GDS-15", "ERQ (reappraisal)", "ERQ (suppression)", "LARSS","Emotional loneliness", "Social loneliness", "BRS"), main = "Coefficients for all cognitive & socio-affective outcomes variables over time per group") + facet_wrap(~variable)
```

## Looking if current depression status differentially affects outcomes

We’ve now seen the differences between non-depressed participants,
and those who had a major depressive episode either now or in the past
25 years. Next, a more exploratory analysis will show if we see
different patterns between those who are currently depressed (either
according to DSM-V criteria, or because they have clinically relevant
symptoms according to the GDS-15) and those who had a depressive episode
in the past

```
# Create a new column that shows whether someone is experiencing clinically relevant dep. symptoms now or whether this happened in the past 25 years
data_melt = data_melt %>% 
  mutate(., group_2 = with(., case_when(
    (group == "(past) depression" & gds_15_score_screening > 4) ~ "current_depression",
    (group == "(past) depression" & scid_5_huidige_stemmingsepisode_screening == "ja") ~ "current_depression",
    (group == "(past) depression" & gds_15_score_screening < 5) ~ "past_depression",
    (group == "(past) depression" & scid_5_huidige_stemmingsepisode_screening == "nee") ~ "past_depression",
    (group == "Control") ~ "no_depression"
  )))

# Run model with all the covariates
m_exploratory <- lmer(value ~ variable*time*group_2 + age + cri + sex + nlv_dart + (1 | PP), data=data_melt) 

# Check if model fit is better with this distinction
anova(m_exploratory, m_complete) # the model without the split has a better fit so we retain this original model.
```

```
## refitting model(s) with ML (instead of REML)
```

```
## Data: data_melt
## Models:
## m_complete: value ~ variable * time * group + age + cri + sex + nlv_dart + (1 | PP)
## m_exploratory: value ~ variable * time * group_2 + age + cri + sex + nlv_dart + (1 | PP)
##               npar    AIC    BIC  logLik deviance  Chisq Df Pr(>Chisq)   
## m_complete     120 5187.9 5853.9 -2473.9   4947.9                        
## m_exploratory  177 5216.9 6199.4 -2431.4   4862.9 84.967 57   0.009559 **
## ---
## Signif. codes:  0 '***' 0.001 '**' 0.01 '*' 0.05 '.' 0.1 ' ' 1
```

# Why did only the moca score increase?

```
# Could it be the version?
check_moca_version = data %>% filter(intervention == "Taal" & (group == "HC" | group == "LLD")) %>% select(PP, time, group, moca_score, moca_versie)

check_moca_version$moca_versie = plyr::mapvalues(check_moca_version$moca_versie, from=c("A (screening)", "A (van screening)", "A (voormeting)", "B (screening)", "B (van screening)", "B (voormeting)", "C (screening)", "C (van screening)"), to = c("A", "A", "A", "B", "B", "B", "C", "C"))

moca_m = lm(moca_score ~ time*group*moca_versie, data=check_moca_version)

data_melt = data_melt %>% 
  mutate(., var_type = with(., case_when(
    (variable == "cfq_totaal") ~ "cognitive",
    (variable == "moca_score") ~ "cognitive",
    (variable == "vat_e_gepaarde_associatie") ~ "cognitive",
    (variable == "vat_e_vrije_reproductie") ~ "cognitive",
    (variable == "symboolsubstitutie") ~ "cognitive",
    (variable == "digit_span_forward") ~ "cognitive",
    (variable == "digit_span_backward") ~ "cognitive",
    (variable == "letter_number_seq") ~ "cognitive",
    (variable == "tmt_a_tijd_in_sec") ~ "cognitive",
    (variable == "tmt_b_tijd_in_sec") ~ "cognitive",
    (variable == "vf_total") ~ "cognitive",
    (variable == "aes_score_self") ~ "wellbeing",
    (variable == "gds_15_score") ~ "wellbeing",
    (variable == "erq_score_cognitive_reappraisal") ~ "wellbeing",
    (variable == "erq_score_expressive_suppression") ~ "wellbeing",
    (variable == "larss_score_totaal") ~ "wellbeing",
    (variable == "loneliness_scale_score_emotional") ~ "wellbeing",
    (variable == "loneliness_scale_score_social") ~ "wellbeing",
    (variable == "brs_score") ~ "wellbeing"
  )))

m_var_type  <- lmer(value ~ var_type*time*group + age + cri + sex + nlv_dart + (1 | PP), data=data_melt)
```

# Language model

## Select variables

We’ve now looked at cognitive and socioaffective measures. However,
we’re also interested in checking if the language group actually
improved in their skills. Since only one group did the English tasks,
and since these were only collected at pre and posttest, we run a
separate model for them. Since the steps are the same we only include
the code.

```
# select columns and select only healthy participants in the language group
data_wide = data %>% select("PP",starts_with(c(
                                       # Covariates
                                       "age",
                                       "group",
                                       "intervention", 
                                       "time",
                                       "cri", 
                                       "av_exp",
                                       "geslacht",                        
                                       "nlv_dart_score_gecorrigeerd_voor_leeftijd_geslacht",
                                       
                                       # English
                                       "ppvt_raw",
                                       "ielts_ls_raw",
                                       "ielts_sp_",
                                       "vf_eng_total",
                                       "ENG_CANDO_PRODUCTIVE",
                                       "ENG_CANDO_RECEPTIVE"
))) %>%  select(-ends_with(c("screening", "uitgestelde_herkenning", "beroep", "vrije_tijd", "opleiding", "informant"))) %>% filter((group == "HC" | group == "LLD") & intervention == "Taal" & time != "followup")


data_wide = droplevels(data_wide) # Drop unused levels

# Make some column names a bit more informative
data_wide = plyr::rename(data_wide, c("geslacht" = "sex",
                                      "nlv_dart_score_gecorrigeerd_voor_leeftijd_geslacht" = "nlv_dart"))

# Z-scale all numerical variables
var_list <- colnames(data_wide[10:16])
data_wide[var_list] <- lapply(data_wide[var_list], function(x) scale(x, center=T))

data_wide = data_wide %>% mutate(group = recode(group, "HC" = "Control", "LLD" = "(past) depression")) # Recode group variable so it is more informative

# Melt dataframe (i.e.,: all DVs are put in one column called Value; another column is added that indicates which test is represented by the Value column)
data_melt <- reshape2::melt(data_wide, id.vars=c("PP", "group","age", "time", "intervention", "cri", "av_exp", "sex", "nlv_dart"))
```

```
## Warning: attributes are not identical across measure variables; they will be
## dropped
```

```
head(data_melt %>% select(starts_with(c("value", "variable", "time", "PP", "intervention")))) # Show first couple of rows to illustrate
```

```
##        value variable    time   PP intervention
## 1  0.4314308 ppvt_raw pretest 1002         Taal
## 2  0.1634971 ppvt_raw pretest 1004         Taal
## 3 -0.6849594 ppvt_raw pretest 1008         Taal
## 4         NA ppvt_raw pretest 1009         Taal
## 5 -1.5780715 ppvt_raw pretest 1011         Taal
## 6  0.1188415 ppvt_raw pretest 1013         Taal
```

## Run analysis and do model check

```
# Run model
m_lang <- lmer(value ~ variable*time*group + age + cri + sex + nlv_dart + (1 | PP), data=data_melt) 

# Check model: seems fine. Not necessary to rerun as GAMM 
par(mfrow=c(2,2))
check_model(m_lang)
```

```
## Variable `Component` is not in your data frame :/
```

## Results: comparing change within and between groups

```
est <- emmeans(m_lang, ~ time*group | variable, adjust="none") # calculate coefficient per task & group

# See if group X has change on score Y over time
within_group <- (pairs(est, simple="time", adjust="none")) 
eff_within <- as.data.frame(eff_size(within_group, sigma=sigma(m_lang), df.residual(m_lang), method="identity")) # calculate Cohen's D
within_group = as.data.frame(within_group)
within_group = cbind(within_group, eff_within[4])

# See if group X has change on score Y at a specific point in time
over_time <- (pairs(est, simple="group", adjust="none"))
eff_over_time <- as.data.frame(eff_size(over_time, sigma=sigma(m_lang), df.residual(m_lang), method="identity")) # calculate Cohen's D
over_time = as.data.frame(over_time)
over_time = cbind(over_time, eff_over_time[4])

# Compare & contrast time effects by group
between_groups <- (pairs(est, interaction="pairwise", adjust="none")) 
eff_between_groups <- as.data.frame(eff_size(between_groups, sigma=sigma(m_lang), df.residual(m_lang), method="identity")) # calculate Cohen's D
between_groups = as.data.frame(between_groups)
between_groups = cbind(between_groups, eff_between_groups[3])

# Show significant differences between group at respective timepoints
over_time %>% filter(p.value < .05) %>% mutate_at(4:7, round,3) %>% mutate_at(8, round,4) %>% mutate_at(9, round,3) %>% kbl() %>% kable_styling()
```

| contrast | time | variable | estimate | SE | df | t.ratio | p.value | effect.size |
| --- | --- | --- | --- | --- | --- | --- | --- | --- |
| Control - (past) depression | pretest | ielts\_ls\_raw | 0.701 | 0.325 | 166.086 | 2.155 | 0.0326 | 0.930 |
| Control - (past) depression | pretest | ielts\_sp\_without\_prep | 0.717 | 0.324 | 164.248 | 2.216 | 0.0280 | 0.952 |
| Control - (past) depression | pretest | ielts\_sp\_with\_prep | 0.645 | 0.326 | 167.948 | 1.978 | 0.0496 | 0.856 |
| Control - (past) depression | posttest | vf\_eng\_total | 0.901 | 0.328 | 170.432 | 2.748 | 0.0067 | 1.196 |

```
# show significant changes over time
within_group %>% filter(p.value < .05) %>% mutate_at(4:7, round,3) %>% mutate_at(8, round,4) %>% mutate_at(9, round,3)  %>% kbl() %>% kable_styling()
```

| contrast | group | variable | estimate | SE | df | t.ratio | p.value | effect.size |
| --- | --- | --- | --- | --- | --- | --- | --- | --- |
| pretest - posttest | (past) depression | ielts\_ls\_raw | -0.774 | 0.294 | 387.042 | -2.631 | 0.0088 | -1.027 |
| pretest - posttest | (past) depression | ielts\_sp\_without\_prep | -0.800 | 0.257 | 386.867 | -3.116 | 0.0020 | -1.062 |
| pretest - posttest | (past) depression | ielts\_sp\_with\_prep | -0.680 | 0.260 | 387.079 | -2.610 | 0.0094 | -0.902 |
| pretest - posttest | Control | vf\_eng\_total | -1.028 | 0.287 | 385.964 | -3.588 | 0.0004 | -1.364 |
| pretest - posttest | (past) depression | vf\_eng\_total | -0.545 | 0.257 | 386.867 | -2.120 | 0.0346 | -0.723 |
| pretest - posttest | Control | ENG\_CANDO\_PRODUCTIVE | -0.903 | 0.275 | 385.281 | -3.279 | 0.0011 | -1.198 |
| pretest - posttest | (past) depression | ENG\_CANDO\_PRODUCTIVE | -0.785 | 0.248 | 385.847 | -3.161 | 0.0017 | -1.042 |
| pretest - posttest | Control | ENG\_CANDO\_RECEPTIVE | -0.661 | 0.275 | 385.281 | -2.399 | 0.0169 | -0.877 |
| pretest - posttest | (past) depression | ENG\_CANDO\_RECEPTIVE | -0.611 | 0.245 | 385.812 | -2.495 | 0.0130 | -0.811 |

```
# Show at which points a change over time differed between groups
between_groups %>% filter(p.value < .05) %>% mutate_at(4:7, round,3) %>% mutate_at(8, round,4) %>% mutate_at(9, round,3) %>% kbl() %>% kable_styling()
```

| time\_pairwise | group\_pairwise | variable | estimate | SE | df | t.ratio | p.value | effect.size |
| --- | --- | --- | --- | --- | --- | --- | --- | --- |
|
|  |

## Plotting results for language outcomes

```
# Plot everything!!!
interactions::cat_plot(m_lang, pred=time, modx = group, mod2=variable, dodge.width = .5, errorbar.width = .33, line.thickness = .85,mod2.labels  =c("PPVT raw", "IELTS listening", "IELTS speaking (without prep)", "IELTS speaking (with prep)", "VF (total)", "Can-do (productive)", "Can-do (receptive)")) + facet_wrap(~variable)
```
