# Supplementary material for: Language Learning as a Non-Pharmacological Intervention in Older Adults with (Past) Depression
Source: Brain Sci. 2025 Sep 15;15(9):991. doi: 10.3390/brainsci15090991 (PMC12468076; doi:10.3390/brainsci15090991)
Supplement: Supplementary file 1 [file brainsci-15-00991-s001.zip › S3 -Complete-results-including-non-significant-findings.html]

Intervention Study - Complete Results including non-significant


# Intervention Study - Complete Results including non-significant

#### Jelle Brouwer, Floor van den Berg, Remco Knooihuizen, Hanneke Loerts, & Merel Keijzer

#### 2025-09-01

## Loading packages

```
library(dplyr) # For more legible code (version 1.1.0)
```

```
## 
## Attaching package: 'dplyr'
```

```
## The following objects are masked from 'package:stats':
## 
##     filter, lag
```

```
## The following objects are masked from 'package:base':
## 
##     intersect, setdiff, setequal, union
```

```
library(reshape2) # For reshaping the DF (version 1.4.4)
library(FSA) # For descriptives (version 0.9.4)
```

```
## ## FSA v0.9.5. See citation('FSA') if used in publication.
## ## Run fishR() for related website and fishR('IFAR') for related book.
```

```
library(lme4) # For running regression (version 1.1-31)
```

```
## Loading required package: Matrix
```

```
library(lmerTest) # For forcing lme4 to give p-values (version 3.1-3)
```

```
## Warning: package 'lmerTest' was built under R version 4.4.3
```

```
## 
## Attaching package: 'lmerTest'
```

```
## The following object is masked from 'package:lme4':
## 
##     lmer
```

```
## The following object is masked from 'package:stats':
## 
##     step
```

```
library(performance) # For model checks (version 0.10.2)
```

```
## Warning: package 'performance' was built under R version 4.4.3
```

```
library(mgcv) # For running regression as GAM (version 1.8-41)
```

```
## Loading required package: nlme
```

```
## 
## Attaching package: 'nlme'
```

```
## The following object is masked from 'package:lme4':
## 
##     lmList
```

```
## The following object is masked from 'package:dplyr':
## 
##     collapse
```

```
## This is mgcv 1.9-1. For overview type 'help("mgcv-package")'.
```

```
library(interactions)
```

```
## Warning: package 'interactions' was built under R version 4.4.2
```

```
library(ggplot2) # For making the plots a bit more interpretable (version 3.4.1)
library(emmeans) # For planned comparisons between groups (version 1.8.4-1)
```

```
## Welcome to emmeans.
## Caution: You lose important information if you filter this package's results.
## See '? untidy'
```

```
library(parameters) # For extracting model parameters (version 0.20.2)
```

```
## Warning: package 'parameters' was built under R version 4.4.3
```

```
library(interactions) # For plotting results (version 1.1.5)

library(knitr) # Make cool looking tables
library(kableExtra)
```

```
## Warning: package 'kableExtra' was built under R version 4.4.3
```

```
## 
## Attaching package: 'kableExtra'
```

```
## The following object is masked from 'package:dplyr':
## 
##     group_rows
```

```
options(scipen=999) # Turn off scientific notation
```

# Cognitive & socio-affective model

## Load data & select variables

We’ll start by loading the raw data. Since the raw data contains a
large number of variables (174, to be precise), we’ll make a subset of
the variables that will be used in the analysis. This will make it much
easier to keep an overview.

```
load(file="C:/Users/u134126/OneDrive - Radboud Universiteit/Ru-Drive/Documents/Diary study/intervention_data_complete_30-01-2023.Rdata") # Load data

# select columns
data_wide = data %>% select("PP",starts_with(c(
                                       # Covariates
                                       "age",
                                       "group",
                                       "intervention", 
                                       "time",
                                       "cri", 
                                       "av_exp",
                                       "geslacht",                        
                                       "nlv_dart_score_gecorrigeerd_voor_leeftijd_geslacht",
                                       "gds_15_score_screening",
                                       "scid_5_huidige_stemmingsepisode_screening",
                                       
                                       # Cognition
                                       "cfq_totaal",                                                     
                                       "moca_score", 
                                       "vat_e_g",
                                       "vat_e_v",
                                       "symboolsubs",
                                       "cijferreeksen_vooruit_aantal_goed",
                                       "cijferreeksen_achteruit_aantal_goed",
                                       "cijfer_letter_reeksen_aantal_goed",
                                       "tmt_a",
                                       "tmt_b_tijd",
                                       "totaal_totaal",
                                       
                                       # Socio-affect / well-being
                                       "aes_score_self",
                                       "gds_15_score",
                                       "erq", 
                                       "larss_score_totaal", 
                                       "loneliness_scale_", 
                                       "brs" 
))) %>%  select(-ends_with(c("moca_score_screening", "moca_versie_screening","nodig_screening", "uitgestelde_herkenning", "beroep", "vrije_tijd", "opleiding", "informant")))  %>% filter((group == "HC" | group == "LLD") & intervention == "Taal")

# Make some column names a bit more informative
data_wide = plyr::rename(data_wide, c("geslacht" = "sex",
                                      "nlv_dart_score_gecorrigeerd_voor_leeftijd_geslacht" = "nlv_dart",
                                      "cijferreeksen_vooruit_aantal_goed" = "digit_span_forward",
                                      "cijferreeksen_achteruit_aantal_goed" = "digit_span_backward",
                                      "cijfer_letter_reeksen_aantal_goed" = "letter_number_seq",
                                      "totaal_totaal_aantal_woorden" = "vf_total"))

data_wide = droplevels(data_wide) # Drop unused levels

data_wide = data_wide %>% mutate(group = recode(group, "HC" = "Control", "LLD" = "(past) depression")) # Recode group variable so it is more informative
```

# Preparing the DF for analysis

For some of the DVs we have, a higher score is negative (e.g., higher
CFQ score means more cognitive failures), for others higher is positive
(e.g., a higher BRS score means someone is more resilient). To make
interpretability easier, we reverse some variables, such that a higher
score always represents a positive score.

After this we will melt the dataframe. In essence, this means putting
the scores (value) for each outcome variable into a single column. A
second column (variable) is added to indicate which task/questionnaire
the score represents. At the end of this block you can see an
illustration of what this looks like.

```
# Reverse where necessary
data_wide$cfq_totaal <- data_wide$cfq_totaal *-1
data_wide$tmt_a_tijd_in_sec <- data_wide$tmt_a_tijd_in_sec *-1
data_wide$tmt_b_tijd_in_sec <- data_wide$tmt_b_tijd_in_sec *-1
data_wide$aes_score_self <- data_wide$aes_score_self *-1
data_wide$gds_15_score <- data_wide$gds_15_score *-1
data_wide$erq_score_expressive_suppression <- data_wide$erq_score_expressive_suppression *-1
data_wide$larss_score_totaal <- data_wide$larss_score_totaal *-1
data_wide$loneliness_scale_score_emotional <- data_wide$loneliness_scale_score_emotional *-1
data_wide$loneliness_scale_score_social <- data_wide$loneliness_scale_score_social *-1

# Z-scale all numerical variables
var_list <- colnames(data_wide[12:30])
data_wide[var_list] <- lapply(data_wide[var_list], function(x) scale(x, center=T))

# Melt dataframe (i.e.,: all DVs are put in one column called Value; another column is added that indicates which test is represented by the Value column)
data_melt <- reshape2::melt(data_wide, id.vars=c("PP", "group","age", "time", "intervention", "cri", "av_exp", "sex", "nlv_dart", "scid_5_huidige_stemmingsepisode_screening", "gds_15_score_screening"))
```

```
## Warning: attributes are not identical across measure variables; they will be
## dropped
```

```
head(data_melt %>% select(starts_with(c("value", "variable", "time", "PP", "intervention")))) # Show first couple of rows to illustrate
```

```
##        value   variable    time   PP intervention
## 1  2.1359441 cfq_totaal pretest 1002         Taal
## 2  0.1506954 cfq_totaal pretest 1004         Taal
## 3  0.8951637 cfq_totaal pretest 1008         Taal
## 4 -0.4283354 cfq_totaal pretest 1009         Taal
## 5  0.7297263 cfq_totaal pretest 1011         Taal
## 6  0.6470076 cfq_totaal pretest 1013         Taal
```

# Run analysis (see appendix B for model checks)

```
# Run model with all the covariates
m_complete <- lmer(value ~ variable*time*group + age + cri + sex + nlv_dart + (1 | PP), data=data_melt)
```

# Results: comparing change within and between groups

we’ve now built our model with cognitive and socio-affective
variables. Next, let’s have a look at the estimates. The
within\_group\_comp DF is used to see if one group improves over time.
Please note that the within\_group\_comp DF looks at ALL comparisons. This
is unnecessary. For instance, we’re not interested in if the GDS score
at follow-up for the music group differs significantly from the pre-test
score for the lecture group, as this comparisons in itself isn’t
interesting (i.e., we need to look at changes over time per group, not
just the raw scores). Therefore, we’ll first use emmeans to run all
comparisons, and then we’ll filter them in DF temp, so that we are left
with the comparisons within each group.

The between group comparisons are made in a different DF. This DF
shows us if a change in a score on a variable over time differs
significantly between groups. Please note that we do not adjust our
p-values, since all comparisons have been planned beforehand. Running
emmeans with an adjustment (e.g., bonferroni) would lead to p-values
that are overly conservative, since it would correct for multiple
comparisons that are made in the code that will not be looked at for the
actual results.

```
est <- emmeans(m_complete, ~time * group | variable, adjust = "none")  # calculate coefficient per task & group

# See if group X has change on score Y over time
within_group <- (pairs(est, simple = "time", adjust = "none"))
eff_within <- as.data.frame(eff_size(within_group, sigma = sigma(m_complete),
    df.residual(m_complete), method = "identity"))  # calculate Cohen's D
within_group = as.data.frame(within_group)
within_group = cbind(within_group, eff_within[4])

# See if group X has change on score Y at a specific point
# in time
over_time <- (pairs(est, simple = "group", adjust = "none"))
eff_over_time <- as.data.frame(eff_size(over_time, sigma = sigma(m_complete),
    df.residual(m_complete), method = "identity"))  # calculate Cohen's D
over_time = as.data.frame(over_time)
over_time = cbind(over_time, eff_over_time[4])

# Compare & contrast time effects by group
between_groups <- (pairs(est, interaction = "pairwise", adjust = "none"))
eff_between_groups <- as.data.frame(eff_size(between_groups,
    sigma = sigma(m_complete), df.residual(m_complete), method = "identity"))  # calculate Cohen's D
between_groups = as.data.frame(between_groups)
between_groups = cbind(between_groups, eff_between_groups[3])

# Show significant differences between group at respective
# timepoints
over_time %>%
    mutate_at(4:7, round, 2) %>%
    mutate_at(8, round, 4) %>%
    mutate_at(9, round, 2) %>%
    kbl() %>%
    kable_styling()
```

| contrast | time | variable | estimate | SE | df | t.ratio | p.value | effect.size |
| --- | --- | --- | --- | --- | --- | --- | --- | --- |
| Control - (past) depression | pretest | cfq\_totaal | 1.22 | 0.33 | 1186.10 | 3.74 | 0.0002 | 1.35 |
| Control - (past) depression | posttest | cfq\_totaal | 0.64 | 0.33 | 1185.69 | 1.97 | 0.0495 | 0.71 |
| Control - (past) depression | followup | cfq\_totaal | 0.62 | 0.33 | 1206.47 | 1.88 | 0.0600 | 0.69 |
| Control - (past) depression | pretest | moca\_score | 0.96 | 0.33 | 1186.10 | 2.95 | 0.0032 | 1.07 |
| Control - (past) depression | posttest | moca\_score | 0.22 | 0.33 | 1185.69 | 0.66 | 0.5091 | 0.24 |
| Control - (past) depression | followup | moca\_score | 0.88 | 0.34 | 1237.97 | 2.63 | 0.0087 | 0.98 |
| Control - (past) depression | pretest | vat\_e\_gepaarde\_associatie | -0.03 | 0.33 | 1186.10 | -0.10 | 0.9198 | -0.04 |
| Control - (past) depression | posttest | vat\_e\_gepaarde\_associatie | -0.04 | 0.33 | 1185.69 | -0.13 | 0.8968 | -0.05 |
| Control - (past) depression | followup | vat\_e\_gepaarde\_associatie | -0.08 | 0.33 | 1206.47 | -0.25 | 0.8033 | -0.09 |
| Control - (past) depression | pretest | vat\_e\_vrije\_reproductie | 0.46 | 0.33 | 1186.10 | 1.42 | 0.1572 | 0.51 |
| Control - (past) depression | posttest | vat\_e\_vrije\_reproductie | 0.36 | 0.33 | 1185.69 | 1.09 | 0.2746 | 0.40 |
| Control - (past) depression | followup | vat\_e\_vrije\_reproductie | 0.48 | 0.33 | 1206.47 | 1.46 | 0.1449 | 0.53 |
| Control - (past) depression | pretest | symboolsubstitutie | 0.44 | 0.35 | 1309.79 | 1.25 | 0.2099 | 0.49 |
| Control - (past) depression | posttest | symboolsubstitutie | 0.42 | 0.34 | 1255.28 | 1.23 | 0.2192 | 0.46 |
| Control - (past) depression | followup | symboolsubstitutie | 0.47 | 0.34 | 1277.28 | 1.38 | 0.1687 | 0.52 |
| Control - (past) depression | pretest | digit\_span\_forward | 0.71 | 0.33 | 1186.10 | 2.16 | 0.0306 | 0.78 |
| Control - (past) depression | posttest | digit\_span\_forward | 0.82 | 0.33 | 1185.69 | 2.52 | 0.0120 | 0.91 |
| Control - (past) depression | followup | digit\_span\_forward | 0.33 | 0.34 | 1237.97 | 0.98 | 0.3261 | 0.37 |
| Control - (past) depression | pretest | digit\_span\_backward | 0.64 | 0.33 | 1186.10 | 1.95 | 0.0508 | 0.71 |
| Control - (past) depression | posttest | digit\_span\_backward | 0.82 | 0.33 | 1185.69 | 2.50 | 0.0125 | 0.90 |
| Control - (past) depression | followup | digit\_span\_backward | 0.40 | 0.34 | 1237.97 | 1.18 | 0.2387 | 0.44 |
| Control - (past) depression | pretest | letter\_number\_seq | 0.56 | 0.33 | 1186.10 | 1.71 | 0.0884 | 0.62 |
| Control - (past) depression | posttest | letter\_number\_seq | 1.34 | 0.33 | 1185.69 | 4.09 | 0.0000 | 1.48 |
| Control - (past) depression | followup | letter\_number\_seq | 0.22 | 0.34 | 1237.97 | 0.64 | 0.5199 | 0.24 |
| Control - (past) depression | pretest | tmt\_a\_tijd\_in\_sec | 0.58 | 0.33 | 1186.10 | 1.78 | 0.0755 | 0.64 |
| Control - (past) depression | posttest | tmt\_a\_tijd\_in\_sec | 0.47 | 0.33 | 1185.69 | 1.44 | 0.1493 | 0.52 |
| Control - (past) depression | followup | tmt\_a\_tijd\_in\_sec | 0.40 | 0.33 | 1206.47 | 1.22 | 0.2231 | 0.45 |
| Control - (past) depression | pretest | tmt\_b\_tijd\_in\_sec | 0.36 | 0.33 | 1186.10 | 1.09 | 0.2739 | 0.40 |
| Control - (past) depression | posttest | tmt\_b\_tijd\_in\_sec | 0.45 | 0.33 | 1185.69 | 1.37 | 0.1702 | 0.50 |
| Control - (past) depression | followup | tmt\_b\_tijd\_in\_sec | 0.18 | 0.33 | 1206.47 | 0.56 | 0.5779 | 0.20 |
| Control - (past) depression | pretest | vf\_total | 0.19 | 0.33 | 1186.10 | 0.58 | 0.5613 | 0.21 |
| Control - (past) depression | posttest | vf\_total | 0.28 | 0.33 | 1185.69 | 0.86 | 0.3887 | 0.31 |
| Control - (past) depression | followup | vf\_total | 0.66 | 0.33 | 1206.47 | 2.01 | 0.0445 | 0.74 |
| Control - (past) depression | pretest | aes\_score\_self | 0.81 | 0.33 | 1186.10 | 2.47 | 0.0138 | 0.89 |
| Control - (past) depression | posttest | aes\_score\_self | -0.10 | 0.33 | 1206.23 | -0.31 | 0.7554 | -0.11 |
| Control - (past) depression | followup | aes\_score\_self | 0.83 | 0.34 | 1277.28 | 2.42 | 0.0158 | 0.92 |
| Control - (past) depression | pretest | gds\_15\_score | 1.19 | 0.33 | 1186.10 | 3.65 | 0.0003 | 1.32 |
| Control - (past) depression | posttest | gds\_15\_score | 0.59 | 0.33 | 1185.69 | 1.79 | 0.0730 | 0.65 |
| Control - (past) depression | followup | gds\_15\_score | 0.64 | 0.33 | 1206.47 | 1.95 | 0.0520 | 0.71 |
| Control - (past) depression | pretest | erq\_score\_cognitive\_reappraisal | 0.47 | 0.33 | 1186.10 | 1.45 | 0.1479 | 0.52 |
| Control - (past) depression | posttest | erq\_score\_cognitive\_reappraisal | 0.10 | 0.33 | 1185.69 | 0.32 | 0.7520 | 0.11 |
| Control - (past) depression | followup | erq\_score\_cognitive\_reappraisal | 0.17 | 0.33 | 1206.47 | 0.52 | 0.6010 | 0.19 |
| Control - (past) depression | pretest | erq\_score\_expressive\_suppression | -0.10 | 0.33 | 1186.10 | -0.30 | 0.7616 | -0.11 |
| Control - (past) depression | posttest | erq\_score\_expressive\_suppression | -0.29 | 0.33 | 1185.69 | -0.89 | 0.3748 | -0.32 |
| Control - (past) depression | followup | erq\_score\_expressive\_suppression | -0.19 | 0.33 | 1206.47 | -0.57 | 0.5681 | -0.21 |
| Control - (past) depression | pretest | larss\_score\_totaal | 0.69 | 0.33 | 1186.10 | 2.10 | 0.0359 | 0.76 |
| Control - (past) depression | posttest | larss\_score\_totaal | 0.89 | 0.33 | 1185.69 | 2.72 | 0.0065 | 0.99 |
| Control - (past) depression | followup | larss\_score\_totaal | 0.69 | 0.33 | 1206.47 | 2.09 | 0.0364 | 0.77 |
| Control - (past) depression | pretest | loneliness\_scale\_score\_emotional | 0.88 | 0.33 | 1186.10 | 2.68 | 0.0074 | 0.97 |
| Control - (past) depression | posttest | loneliness\_scale\_score\_emotional | 0.76 | 0.33 | 1185.69 | 2.33 | 0.0200 | 0.84 |
| Control - (past) depression | followup | loneliness\_scale\_score\_emotional | 0.89 | 0.33 | 1206.47 | 2.69 | 0.0072 | 0.98 |
| Control - (past) depression | pretest | loneliness\_scale\_score\_social | 1.37 | 0.33 | 1186.10 | 4.19 | 0.0000 | 1.52 |
| Control - (past) depression | posttest | loneliness\_scale\_score\_social | 0.75 | 0.33 | 1185.69 | 2.30 | 0.0215 | 0.83 |
| Control - (past) depression | followup | loneliness\_scale\_score\_social | 0.61 | 0.33 | 1206.47 | 1.85 | 0.0647 | 0.68 |
| Control - (past) depression | pretest | brs\_score | 1.27 | 0.33 | 1186.10 | 3.89 | 0.0001 | 1.41 |
| Control - (past) depression | posttest | brs\_score | 0.95 | 0.33 | 1185.69 | 2.91 | 0.0037 | 1.05 |
| Control - (past) depression | followup | brs\_score | 1.06 | 0.33 | 1206.47 | 3.20 | 0.0014 | 1.17 |

```
# show significant changes over time
within_group %>%
    mutate_at(4:7, round, 2) %>%
    mutate_at(8, round, 4) %>%
    mutate_at(9, round, 2) %>%
    kbl() %>%
    kable_styling()
```

| contrast | group | variable | estimate | SE | df | t.ratio | p.value | effect.size |
| --- | --- | --- | --- | --- | --- | --- | --- | --- |
| pretest - posttest | Control | cfq\_totaal | 0.05 | 0.33 | 1757.11 | 0.15 | 0.8834 | 0.05 |
| pretest - followup | Control | cfq\_totaal | -0.12 | 0.33 | 1759.17 | -0.36 | 0.7213 | -0.13 |
| posttest - followup | Control | cfq\_totaal | -0.17 | 0.33 | 1756.45 | -0.50 | 0.6146 | -0.18 |
| pretest - posttest | (past) depression | cfq\_totaal | -0.53 | 0.29 | 1757.59 | -1.81 | 0.0697 | -0.59 |
| pretest - followup | (past) depression | cfq\_totaal | -0.72 | 0.30 | 1759.98 | -2.42 | 0.0157 | -0.80 |
| posttest - followup | (past) depression | cfq\_totaal | -0.19 | 0.30 | 1756.87 | -0.63 | 0.5298 | -0.21 |
| pretest - posttest | Control | moca\_score | -0.08 | 0.33 | 1757.11 | -0.25 | 0.8039 | -0.09 |
| pretest - followup | Control | moca\_score | -0.36 | 0.34 | 1759.39 | -1.07 | 0.2865 | -0.40 |
| posttest - followup | Control | moca\_score | -0.28 | 0.34 | 1756.77 | -0.82 | 0.4110 | -0.31 |
| pretest - posttest | (past) depression | moca\_score | -0.83 | 0.29 | 1757.59 | -2.83 | 0.0047 | -0.92 |
| pretest - followup | (past) depression | moca\_score | -0.44 | 0.30 | 1759.98 | -1.48 | 0.1403 | -0.49 |
| posttest - followup | (past) depression | moca\_score | 0.39 | 0.30 | 1756.87 | 1.32 | 0.1887 | 0.43 |
| pretest - posttest | Control | vat\_e\_gepaarde\_associatie | -0.51 | 0.33 | 1757.11 | -1.54 | 0.1245 | -0.56 |
| pretest - followup | Control | vat\_e\_gepaarde\_associatie | -0.80 | 0.33 | 1759.17 | -2.43 | 0.0152 | -0.89 |
| posttest - followup | Control | vat\_e\_gepaarde\_associatie | -0.29 | 0.33 | 1756.45 | -0.89 | 0.3721 | -0.33 |
| pretest - posttest | (past) depression | vat\_e\_gepaarde\_associatie | -0.52 | 0.29 | 1757.59 | -1.76 | 0.0782 | -0.57 |
| pretest - followup | (past) depression | vat\_e\_gepaarde\_associatie | -0.85 | 0.30 | 1759.98 | -2.86 | 0.0043 | -0.94 |
| posttest - followup | (past) depression | vat\_e\_gepaarde\_associatie | -0.33 | 0.30 | 1756.87 | -1.13 | 0.2607 | -0.37 |
| pretest - posttest | Control | vat\_e\_vrije\_reproductie | -0.42 | 0.33 | 1757.11 | -1.28 | 0.1994 | -0.47 |
| pretest - followup | Control | vat\_e\_vrije\_reproductie | -0.56 | 0.33 | 1759.17 | -1.71 | 0.0882 | -0.62 |
| posttest - followup | Control | vat\_e\_vrije\_reproductie | -0.14 | 0.33 | 1756.45 | -0.42 | 0.6723 | -0.15 |
| pretest - posttest | (past) depression | vat\_e\_vrije\_reproductie | -0.53 | 0.29 | 1757.59 | -1.80 | 0.0714 | -0.59 |
| pretest - followup | (past) depression | vat\_e\_vrije\_reproductie | -0.54 | 0.30 | 1759.98 | -1.83 | 0.0676 | -0.60 |
| posttest - followup | (past) depression | vat\_e\_vrije\_reproductie | -0.01 | 0.30 | 1756.87 | -0.05 | 0.9601 | -0.02 |
| pretest - posttest | Control | symboolsubstitutie | -0.26 | 0.36 | 1759.25 | -0.72 | 0.4725 | -0.29 |
| pretest - followup | Control | symboolsubstitutie | -0.38 | 0.35 | 1760.02 | -1.10 | 0.2735 | -0.42 |
| posttest - followup | Control | symboolsubstitutie | -0.12 | 0.34 | 1757.02 | -0.36 | 0.7184 | -0.14 |
| pretest - posttest | (past) depression | symboolsubstitutie | -0.28 | 0.30 | 1758.00 | -0.95 | 0.3415 | -0.31 |
| pretest - followup | (past) depression | symboolsubstitutie | -0.35 | 0.32 | 1760.79 | -1.11 | 0.2684 | -0.39 |
| posttest - followup | (past) depression | symboolsubstitutie | -0.07 | 0.31 | 1757.73 | -0.22 | 0.8292 | -0.07 |
| pretest - posttest | Control | digit\_span\_forward | -0.21 | 0.33 | 1757.11 | -0.63 | 0.5263 | -0.23 |
| pretest - followup | Control | digit\_span\_forward | 0.32 | 0.34 | 1759.39 | 0.95 | 0.3431 | 0.35 |
| posttest - followup | Control | digit\_span\_forward | 0.53 | 0.34 | 1756.77 | 1.57 | 0.1163 | 0.58 |
| pretest - posttest | (past) depression | digit\_span\_forward | -0.09 | 0.29 | 1757.59 | -0.32 | 0.7480 | -0.10 |
| pretest - followup | (past) depression | digit\_span\_forward | -0.06 | 0.30 | 1759.98 | -0.20 | 0.8447 | -0.06 |
| posttest - followup | (past) depression | digit\_span\_forward | 0.04 | 0.30 | 1756.87 | 0.12 | 0.9038 | 0.04 |
| pretest - posttest | Control | digit\_span\_backward | -0.36 | 0.33 | 1757.11 | -1.09 | 0.2760 | -0.40 |
| pretest - followup | Control | digit\_span\_backward | 0.11 | 0.34 | 1759.39 | 0.31 | 0.7529 | 0.12 |
| posttest - followup | Control | digit\_span\_backward | 0.47 | 0.34 | 1756.77 | 1.39 | 0.1661 | 0.52 |
| pretest - posttest | (past) depression | digit\_span\_backward | -0.18 | 0.29 | 1757.59 | -0.62 | 0.5381 | -0.20 |
| pretest - followup | (past) depression | digit\_span\_backward | -0.14 | 0.30 | 1759.98 | -0.46 | 0.6462 | -0.15 |
| posttest - followup | (past) depression | digit\_span\_backward | 0.04 | 0.30 | 1756.87 | 0.15 | 0.8823 | 0.05 |
| pretest - posttest | Control | letter\_number\_seq | -1.00 | 0.33 | 1757.11 | -3.02 | 0.0025 | -1.10 |
| pretest - followup | Control | letter\_number\_seq | 0.24 | 0.34 | 1759.39 | 0.70 | 0.4844 | 0.26 |
| posttest - followup | Control | letter\_number\_seq | 1.23 | 0.34 | 1756.77 | 3.67 | 0.0003 | 1.36 |
| pretest - posttest | (past) depression | letter\_number\_seq | -0.22 | 0.29 | 1757.59 | -0.74 | 0.4594 | -0.24 |
| pretest - followup | (past) depression | letter\_number\_seq | -0.11 | 0.30 | 1759.98 | -0.36 | 0.7222 | -0.12 |
| posttest - followup | (past) depression | letter\_number\_seq | 0.11 | 0.30 | 1756.87 | 0.37 | 0.7083 | 0.12 |
| pretest - posttest | Control | tmt\_a\_tijd\_in\_sec | -0.19 | 0.33 | 1757.11 | -0.57 | 0.5683 | -0.21 |
| pretest - followup | Control | tmt\_a\_tijd\_in\_sec | -0.16 | 0.33 | 1759.17 | -0.47 | 0.6367 | -0.17 |
| posttest - followup | Control | tmt\_a\_tijd\_in\_sec | 0.03 | 0.33 | 1756.45 | 0.10 | 0.9218 | 0.04 |
| pretest - posttest | (past) depression | tmt\_a\_tijd\_in\_sec | -0.30 | 0.29 | 1757.59 | -1.02 | 0.3096 | -0.33 |
| pretest - followup | (past) depression | tmt\_a\_tijd\_in\_sec | -0.33 | 0.30 | 1759.98 | -1.12 | 0.2610 | -0.37 |
| posttest - followup | (past) depression | tmt\_a\_tijd\_in\_sec | -0.04 | 0.30 | 1756.87 | -0.12 | 0.9026 | -0.04 |
| pretest - posttest | Control | tmt\_b\_tijd\_in\_sec | 0.04 | 0.33 | 1757.11 | 0.12 | 0.9072 | 0.04 |
| pretest - followup | Control | tmt\_b\_tijd\_in\_sec | -0.19 | 0.33 | 1759.17 | -0.58 | 0.5646 | -0.21 |
| posttest - followup | Control | tmt\_b\_tijd\_in\_sec | -0.23 | 0.33 | 1756.45 | -0.69 | 0.4885 | -0.25 |
| pretest - posttest | (past) depression | tmt\_b\_tijd\_in\_sec | 0.13 | 0.29 | 1757.59 | 0.44 | 0.6594 | 0.14 |
| pretest - followup | (past) depression | tmt\_b\_tijd\_in\_sec | -0.36 | 0.30 | 1759.98 | -1.22 | 0.2212 | -0.40 |
| posttest - followup | (past) depression | tmt\_b\_tijd\_in\_sec | -0.49 | 0.30 | 1756.87 | -1.66 | 0.0973 | -0.55 |
| pretest - posttest | Control | vf\_total | -0.06 | 0.33 | 1757.11 | -0.19 | 0.8525 | -0.07 |
| pretest - followup | Control | vf\_total | -0.58 | 0.33 | 1759.17 | -1.75 | 0.0802 | -0.64 |
| posttest - followup | Control | vf\_total | -0.52 | 0.33 | 1756.45 | -1.57 | 0.1177 | -0.57 |
| pretest - posttest | (past) depression | vf\_total | 0.03 | 0.29 | 1757.59 | 0.10 | 0.9171 | 0.03 |
| pretest - followup | (past) depression | vf\_total | -0.10 | 0.30 | 1759.98 | -0.35 | 0.7282 | -0.11 |
| posttest - followup | (past) depression | vf\_total | -0.13 | 0.30 | 1756.87 | -0.45 | 0.6525 | -0.15 |
| pretest - posttest | Control | aes\_score\_self | 0.32 | 0.33 | 1757.11 | 0.95 | 0.3397 | 0.35 |
| pretest - followup | Control | aes\_score\_self | -0.23 | 0.33 | 1759.17 | -0.69 | 0.4874 | -0.25 |
| posttest - followup | Control | aes\_score\_self | -0.54 | 0.33 | 1756.45 | -1.65 | 0.0991 | -0.60 |
| pretest - posttest | (past) depression | aes\_score\_self | -0.59 | 0.30 | 1757.76 | -2.00 | 0.0459 | -0.66 |
| pretest - followup | (past) depression | aes\_score\_self | -0.20 | 0.31 | 1760.28 | -0.65 | 0.5142 | -0.23 |
| posttest - followup | (past) depression | aes\_score\_self | 0.39 | 0.32 | 1757.48 | 1.23 | 0.2175 | 0.43 |
| pretest - posttest | Control | gds\_15\_score | 0.21 | 0.33 | 1757.11 | 0.64 | 0.5244 | 0.23 |
| pretest - followup | Control | gds\_15\_score | 0.18 | 0.33 | 1759.17 | 0.54 | 0.5907 | 0.20 |
| posttest - followup | Control | gds\_15\_score | -0.03 | 0.33 | 1756.45 | -0.10 | 0.9214 | -0.04 |
| pretest - posttest | (past) depression | gds\_15\_score | -0.40 | 0.29 | 1757.59 | -1.35 | 0.1783 | -0.44 |
| pretest - followup | (past) depression | gds\_15\_score | -0.37 | 0.30 | 1759.98 | -1.25 | 0.2124 | -0.41 |
| posttest - followup | (past) depression | gds\_15\_score | 0.02 | 0.30 | 1756.87 | 0.08 | 0.9362 | 0.03 |
| pretest - posttest | Control | erq\_score\_cognitive\_reappraisal | 0.23 | 0.33 | 1757.11 | 0.68 | 0.4944 | 0.25 |
| pretest - followup | Control | erq\_score\_cognitive\_reappraisal | 0.52 | 0.33 | 1759.17 | 1.58 | 0.1136 | 0.58 |
| posttest - followup | Control | erq\_score\_cognitive\_reappraisal | 0.30 | 0.33 | 1756.45 | 0.90 | 0.3682 | 0.33 |
| pretest - posttest | (past) depression | erq\_score\_cognitive\_reappraisal | -0.14 | 0.29 | 1757.59 | -0.49 | 0.6228 | -0.16 |
| pretest - followup | (past) depression | erq\_score\_cognitive\_reappraisal | 0.22 | 0.30 | 1759.98 | 0.75 | 0.4551 | 0.25 |
| posttest - followup | (past) depression | erq\_score\_cognitive\_reappraisal | 0.37 | 0.30 | 1756.87 | 1.23 | 0.2178 | 0.41 |
| pretest - posttest | Control | erq\_score\_expressive\_suppression | 0.08 | 0.33 | 1757.11 | 0.23 | 0.8166 | 0.08 |
| pretest - followup | Control | erq\_score\_expressive\_suppression | 0.11 | 0.33 | 1759.17 | 0.32 | 0.7502 | 0.12 |
| posttest - followup | Control | erq\_score\_expressive\_suppression | 0.03 | 0.33 | 1756.45 | 0.09 | 0.9310 | 0.03 |
| pretest - posttest | (past) depression | erq\_score\_expressive\_suppression | -0.11 | 0.29 | 1757.59 | -0.39 | 0.6965 | -0.13 |
| pretest - followup | (past) depression | erq\_score\_expressive\_suppression | 0.02 | 0.30 | 1759.98 | 0.05 | 0.9580 | 0.02 |
| posttest - followup | (past) depression | erq\_score\_expressive\_suppression | 0.13 | 0.30 | 1756.87 | 0.44 | 0.6619 | 0.14 |
| pretest - posttest | Control | larss\_score\_totaal | -0.29 | 0.33 | 1757.11 | -0.88 | 0.3771 | -0.32 |
| pretest - followup | Control | larss\_score\_totaal | 0.01 | 0.33 | 1759.17 | 0.04 | 0.9712 | 0.01 |
| posttest - followup | Control | larss\_score\_totaal | 0.30 | 0.33 | 1756.45 | 0.92 | 0.3579 | 0.34 |
| pretest - posttest | (past) depression | larss\_score\_totaal | -0.09 | 0.29 | 1757.59 | -0.30 | 0.7656 | -0.10 |
| pretest - followup | (past) depression | larss\_score\_totaal | 0.02 | 0.30 | 1759.98 | 0.06 | 0.9530 | 0.02 |
| posttest - followup | (past) depression | larss\_score\_totaal | 0.11 | 0.30 | 1756.87 | 0.35 | 0.7240 | 0.12 |
| pretest - posttest | Control | loneliness\_scale\_score\_emotional | 0.05 | 0.33 | 1757.11 | 0.14 | 0.8884 | 0.05 |
| pretest - followup | Control | loneliness\_scale\_score\_emotional | 0.03 | 0.33 | 1759.17 | 0.10 | 0.9168 | 0.04 |
| posttest - followup | Control | loneliness\_scale\_score\_emotional | -0.01 | 0.33 | 1756.45 | -0.04 | 0.9714 | -0.01 |
| pretest - posttest | (past) depression | loneliness\_scale\_score\_emotional | -0.07 | 0.29 | 1757.59 | -0.24 | 0.8134 | -0.08 |
| pretest - followup | (past) depression | loneliness\_scale\_score\_emotional | 0.05 | 0.30 | 1759.98 | 0.16 | 0.8748 | 0.05 |
| posttest - followup | (past) depression | loneliness\_scale\_score\_emotional | 0.12 | 0.30 | 1756.87 | 0.39 | 0.6962 | 0.13 |
| pretest - posttest | Control | loneliness\_scale\_score\_social | -0.02 | 0.33 | 1757.11 | -0.05 | 0.9571 | -0.02 |
| pretest - followup | Control | loneliness\_scale\_score\_social | 0.14 | 0.33 | 1759.17 | 0.42 | 0.6762 | 0.15 |
| posttest - followup | Control | loneliness\_scale\_score\_social | 0.16 | 0.33 | 1756.45 | 0.47 | 0.6372 | 0.17 |
| pretest - posttest | (past) depression | loneliness\_scale\_score\_social | -0.64 | 0.29 | 1757.59 | -2.17 | 0.0303 | -0.70 |
| pretest - followup | (past) depression | loneliness\_scale\_score\_social | -0.62 | 0.30 | 1759.98 | -2.09 | 0.0367 | -0.69 |
| posttest - followup | (past) depression | loneliness\_scale\_score\_social | 0.01 | 0.30 | 1756.87 | 0.05 | 0.9627 | 0.02 |
| pretest - posttest | Control | brs\_score | 0.32 | 0.33 | 1757.11 | 0.96 | 0.3368 | 0.35 |
| pretest - followup | Control | brs\_score | 0.15 | 0.33 | 1759.17 | 0.46 | 0.6430 | 0.17 |
| posttest - followup | Control | brs\_score | -0.16 | 0.33 | 1756.45 | -0.50 | 0.6192 | -0.18 |
| pretest - posttest | (past) depression | brs\_score | 0.00 | 0.29 | 1757.59 | -0.02 | 0.9880 | 0.00 |
| pretest - followup | (past) depression | brs\_score | -0.06 | 0.30 | 1759.98 | -0.21 | 0.8356 | -0.07 |
| posttest - followup | (past) depression | brs\_score | -0.06 | 0.30 | 1756.87 | -0.19 | 0.8471 | -0.06 |

```
# Show at which points a change over time differed between
# groups
between_groups %>%
    mutate_at(4:7, round, 2) %>%
    mutate_at(8, round, 4) %>%
    mutate_at(9, round, 2) %>%
    kbl() %>%
    kable_styling()
```

| time\_pairwise | group\_pairwise | variable | estimate | SE | df | t.ratio | p.value | effect.size |
| --- | --- | --- | --- | --- | --- | --- | --- | --- |
| pretest - posttest | Control - (past) depression | cfq\_totaal | 0.58 | 0.44 | 1755.92 | 1.32 | 0.1886 | 0.64 |
| pretest - followup | Control - (past) depression | cfq\_totaal | 0.60 | 0.44 | 1756.18 | 1.35 | 0.1760 | 0.67 |
| posttest - followup | Control - (past) depression | cfq\_totaal | 0.02 | 0.44 | 1756.19 | 0.05 | 0.9629 | 0.02 |
| pretest - posttest | Control - (past) depression | moca\_score | 0.75 | 0.44 | 1755.92 | 1.69 | 0.0904 | 0.83 |
| pretest - followup | Control - (past) depression | moca\_score | 0.08 | 0.45 | 1756.39 | 0.18 | 0.8575 | 0.09 |
| posttest - followup | Control - (past) depression | moca\_score | -0.67 | 0.45 | 1756.40 | -1.49 | 0.1371 | -0.74 |
| pretest - posttest | Control - (past) depression | vat\_e\_gepaarde\_associatie | 0.01 | 0.44 | 1755.92 | 0.02 | 0.9828 | 0.01 |
| pretest - followup | Control - (past) depression | vat\_e\_gepaarde\_associatie | 0.05 | 0.44 | 1756.18 | 0.11 | 0.9114 | 0.05 |
| posttest - followup | Control - (past) depression | vat\_e\_gepaarde\_associatie | 0.04 | 0.44 | 1756.19 | 0.09 | 0.9284 | 0.04 |
| pretest - posttest | Control - (past) depression | vat\_e\_vrije\_reproductie | 0.11 | 0.44 | 1755.92 | 0.24 | 0.8113 | 0.12 |
| pretest - followup | Control - (past) depression | vat\_e\_vrije\_reproductie | -0.02 | 0.44 | 1756.18 | -0.04 | 0.9652 | -0.02 |
| posttest - followup | Control - (past) depression | vat\_e\_vrije\_reproductie | -0.12 | 0.44 | 1756.19 | -0.28 | 0.7789 | -0.14 |
| pretest - posttest | Control - (past) depression | symboolsubstitutie | 0.02 | 0.47 | 1757.24 | 0.05 | 0.9618 | 0.02 |
| pretest - followup | Control - (past) depression | symboolsubstitutie | -0.03 | 0.47 | 1757.39 | -0.07 | 0.9432 | -0.04 |
| posttest - followup | Control - (past) depression | symboolsubstitutie | -0.06 | 0.46 | 1757.08 | -0.12 | 0.9036 | -0.06 |
| pretest - posttest | Control - (past) depression | digit\_span\_forward | -0.11 | 0.44 | 1755.92 | -0.26 | 0.7946 | -0.13 |
| pretest - followup | Control - (past) depression | digit\_span\_forward | 0.38 | 0.45 | 1756.39 | 0.84 | 0.4008 | 0.42 |
| posttest - followup | Control - (past) depression | digit\_span\_forward | 0.49 | 0.45 | 1756.40 | 1.10 | 0.2730 | 0.54 |
| pretest - posttest | Control - (past) depression | digit\_span\_backward | -0.18 | 0.44 | 1755.92 | -0.41 | 0.6851 | -0.20 |
| pretest - followup | Control - (past) depression | digit\_span\_backward | 0.24 | 0.45 | 1756.39 | 0.54 | 0.5891 | 0.27 |
| posttest - followup | Control - (past) depression | digit\_span\_backward | 0.42 | 0.45 | 1756.40 | 0.94 | 0.3477 | 0.47 |
| pretest - posttest | Control - (past) depression | letter\_number\_seq | -0.78 | 0.44 | 1755.92 | -1.77 | 0.0772 | -0.86 |
| pretest - followup | Control - (past) depression | letter\_number\_seq | 0.34 | 0.45 | 1756.39 | 0.76 | 0.4475 | 0.38 |
| posttest - followup | Control - (past) depression | letter\_number\_seq | 1.12 | 0.45 | 1756.40 | 2.50 | 0.0125 | 1.24 |
| pretest - posttest | Control - (past) depression | tmt\_a\_tijd\_in\_sec | 0.11 | 0.44 | 1755.92 | 0.25 | 0.8038 | 0.12 |
| pretest - followup | Control - (past) depression | tmt\_a\_tijd\_in\_sec | 0.18 | 0.44 | 1756.18 | 0.40 | 0.6878 | 0.20 |
| posttest - followup | Control - (past) depression | tmt\_a\_tijd\_in\_sec | 0.07 | 0.44 | 1756.19 | 0.15 | 0.8769 | 0.08 |
| pretest - posttest | Control - (past) depression | tmt\_b\_tijd\_in\_sec | -0.09 | 0.44 | 1755.92 | -0.21 | 0.8371 | -0.10 |
| pretest - followup | Control - (past) depression | tmt\_b\_tijd\_in\_sec | 0.17 | 0.44 | 1756.18 | 0.39 | 0.6956 | 0.19 |
| posttest - followup | Control - (past) depression | tmt\_b\_tijd\_in\_sec | 0.26 | 0.44 | 1756.19 | 0.60 | 0.5515 | 0.29 |
| pretest - posttest | Control - (past) depression | vf\_total | -0.09 | 0.44 | 1755.92 | -0.21 | 0.8351 | -0.10 |
| pretest - followup | Control - (past) depression | vf\_total | -0.47 | 0.44 | 1756.18 | -1.07 | 0.2855 | -0.53 |
| posttest - followup | Control - (past) depression | vf\_total | -0.38 | 0.44 | 1756.19 | -0.86 | 0.3891 | -0.42 |
| pretest - posttest | Control - (past) depression | aes\_score\_self | 0.91 | 0.44 | 1756.07 | 2.05 | 0.0408 | 1.01 |
| pretest - followup | Control - (past) depression | aes\_score\_self | -0.03 | 0.45 | 1756.69 | -0.06 | 0.9554 | -0.03 |
| posttest - followup | Control - (past) depression | aes\_score\_self | -0.93 | 0.46 | 1756.56 | -2.04 | 0.0410 | -1.03 |
| pretest - posttest | Control - (past) depression | gds\_15\_score | 0.61 | 0.44 | 1755.92 | 1.37 | 0.1707 | 0.67 |
| pretest - followup | Control - (past) depression | gds\_15\_score | 0.55 | 0.44 | 1756.18 | 1.24 | 0.2169 | 0.61 |
| posttest - followup | Control - (past) depression | gds\_15\_score | -0.06 | 0.44 | 1756.19 | -0.13 | 0.8990 | -0.06 |
| pretest - posttest | Control - (past) depression | erq\_score\_cognitive\_reappraisal | 0.37 | 0.44 | 1755.92 | 0.84 | 0.4022 | 0.41 |
| pretest - followup | Control - (past) depression | erq\_score\_cognitive\_reappraisal | 0.30 | 0.44 | 1756.18 | 0.68 | 0.4990 | 0.33 |
| posttest - followup | Control - (past) depression | erq\_score\_cognitive\_reappraisal | -0.07 | 0.44 | 1756.19 | -0.16 | 0.8756 | -0.08 |
| pretest - posttest | Control - (past) depression | erq\_score\_expressive\_suppression | 0.19 | 0.44 | 1755.92 | 0.43 | 0.6654 | 0.21 |
| pretest - followup | Control - (past) depression | erq\_score\_expressive\_suppression | 0.09 | 0.44 | 1756.18 | 0.20 | 0.8403 | 0.10 |
| posttest - followup | Control - (past) depression | erq\_score\_expressive\_suppression | -0.10 | 0.44 | 1756.19 | -0.23 | 0.8193 | -0.11 |
| pretest - posttest | Control - (past) depression | larss\_score\_totaal | -0.20 | 0.44 | 1755.92 | -0.46 | 0.6439 | -0.23 |
| pretest - followup | Control - (past) depression | larss\_score\_totaal | -0.01 | 0.44 | 1756.18 | -0.01 | 0.9899 | -0.01 |
| posttest - followup | Control - (past) depression | larss\_score\_totaal | 0.20 | 0.44 | 1756.19 | 0.45 | 0.6550 | 0.22 |
| pretest - posttest | Control - (past) depression | loneliness\_scale\_score\_emotional | 0.12 | 0.44 | 1755.92 | 0.26 | 0.7935 | 0.13 |
| pretest - followup | Control - (past) depression | loneliness\_scale\_score\_emotional | -0.01 | 0.44 | 1756.18 | -0.03 | 0.9777 | -0.01 |
| posttest - followup | Control - (past) depression | loneliness\_scale\_score\_emotional | -0.13 | 0.44 | 1756.19 | -0.29 | 0.7733 | -0.14 |
| pretest - posttest | Control - (past) depression | loneliness\_scale\_score\_social | 0.62 | 0.44 | 1755.92 | 1.40 | 0.1617 | 0.68 |
| pretest - followup | Control - (past) depression | loneliness\_scale\_score\_social | 0.76 | 0.44 | 1756.18 | 1.71 | 0.0874 | 0.84 |
| posttest - followup | Control - (past) depression | loneliness\_scale\_score\_social | 0.14 | 0.44 | 1756.19 | 0.32 | 0.7497 | 0.16 |
| pretest - posttest | Control - (past) depression | brs\_score | 0.32 | 0.44 | 1755.92 | 0.73 | 0.4665 | 0.36 |
| pretest - followup | Control - (past) depression | brs\_score | 0.21 | 0.44 | 1756.18 | 0.48 | 0.6287 | 0.24 |
| posttest - followup | Control - (past) depression | brs\_score | -0.11 | 0.44 | 1756.19 | -0.24 | 0.8102 | -0.12 |

# Language model

## Select variables

We’ve now looked at cognitive and socioaffective measures. However,
we’re also interested in checking if the language group actually
improved in their skills. Since only one group did the English tasks,
and since these were only collected at pre and posttest, we run a
separate model for them. Since the steps are the same we only include
the code.

```
# select columns and select only healthy participants in the language group
data_wide = data %>% select("PP",starts_with(c(
                                       # Covariates
                                       "age",
                                       "group",
                                       "intervention", 
                                       "time",
                                       "cri", 
                                       "av_exp",
                                       "geslacht",                        
                                       "nlv_dart_score_gecorrigeerd_voor_leeftijd_geslacht",
                                       
                                       # English
                                       "ppvt_raw",
                                       "ielts_ls_raw",
                                       "ielts_sp_",
                                       "vf_eng_total",
                                       "ENG_CANDO_PRODUCTIVE",
                                       "ENG_CANDO_RECEPTIVE"
))) %>%  select(-ends_with(c("screening", "uitgestelde_herkenning", "beroep", "vrije_tijd", "opleiding", "informant"))) %>% filter((group == "HC" | group == "LLD") & intervention == "Taal" & time != "followup")


data_wide = droplevels(data_wide) # Drop unused levels

# Make some column names a bit more informative
data_wide = plyr::rename(data_wide, c("geslacht" = "sex",
                                      "nlv_dart_score_gecorrigeerd_voor_leeftijd_geslacht" = "nlv_dart"))

# Z-scale all numerical variables
var_list <- colnames(data_wide[10:16])
data_wide[var_list] <- lapply(data_wide[var_list], function(x) scale(x, center=T))

data_wide = data_wide %>% mutate(group = recode(group, "HC" = "Control", "LLD" = "(past) depression")) # Recode group variable so it is more informative

# Melt dataframe (i.e.,: all DVs are put in one column called Value; another column is added that indicates which test is represented by the Value column)
data_melt <- reshape2::melt(data_wide, id.vars=c("PP", "group","age", "time", "intervention", "cri", "av_exp", "sex", "nlv_dart"))
```

```
## Warning: attributes are not identical across measure variables; they will be
## dropped
```

```
# See head of DF
head(data_melt %>% select(starts_with(c("value", "variable", "time", "PP", "intervention")))) # Show first couple of rows to illustrate
```

```
##        value variable    time   PP intervention
## 1  0.4314308 ppvt_raw pretest 1002         Taal
## 2  0.1634971 ppvt_raw pretest 1004         Taal
## 3 -0.6849594 ppvt_raw pretest 1008         Taal
## 4         NA ppvt_raw pretest 1009         Taal
## 5 -1.5780715 ppvt_raw pretest 1011         Taal
## 6  0.1188415 ppvt_raw pretest 1013         Taal
```

# Run analysis (see Appendix B for model check)

```
# Run model
m_lang <- lmer(value ~ variable*time*group + age + cri + sex + nlv_dart + (1 | PP), data=data_melt)
```

# Results: comparing change within and between groups

```
est <- emmeans(m_lang, ~ time*group | variable, adjust="none") # calculate coefficient per task & group

# See if group X has change on score Y over time
within_group <- (pairs(est, simple="time", adjust="none")) 
eff_within <- as.data.frame(eff_size(within_group, sigma=sigma(m_lang), df.residual(m_lang), method="identity")) # calculate Cohen's D
within_group = as.data.frame(within_group)
within_group = cbind(within_group, eff_within[4])

# See if group X has change on score Y at a specific point in time
over_time <- (pairs(est, simple="group", adjust="none"))
eff_over_time <- as.data.frame(eff_size(over_time, sigma=sigma(m_lang), df.residual(m_lang), method="identity")) # calculate Cohen's D
over_time = as.data.frame(over_time)
over_time = cbind(over_time, eff_over_time[4])

# Compare & contrast time effects by group
between_groups <- (pairs(est, interaction="pairwise", adjust="none")) 
eff_between_groups <- as.data.frame(eff_size(between_groups, sigma=sigma(m_lang), df.residual(m_lang), method="identity")) # calculate Cohen's D
between_groups = as.data.frame(between_groups)
between_groups = cbind(between_groups, eff_between_groups[3])

# Show significant differences between group at respective timepoints
over_time %>% mutate_at(4:7, round,3) %>% mutate_at(8, round,4) %>% mutate_at(9, round,3) %>% kbl() %>% kable_styling()
```

| contrast | time | variable | estimate | SE | df | t.ratio | p.value | effect.size |
| --- | --- | --- | --- | --- | --- | --- | --- | --- |
| Control - (past) depression | pretest | ppvt\_raw | 0.406 | 0.335 | 180.195 | 1.212 | 0.2269 | 0.539 |
| Control - (past) depression | posttest | ppvt\_raw | 0.417 | 0.328 | 170.432 | 1.272 | 0.2051 | 0.554 |
| Control - (past) depression | pretest | ielts\_ls\_raw | 0.701 | 0.325 | 166.086 | 2.155 | 0.0326 | 0.930 |
| Control - (past) depression | posttest | ielts\_ls\_raw | 0.284 | 0.357 | 212.363 | 0.795 | 0.4275 | 0.377 |
| Control - (past) depression | pretest | ielts\_sp\_without\_prep | 0.717 | 0.324 | 164.248 | 2.216 | 0.0280 | 0.952 |
| Control - (past) depression | posttest | ielts\_sp\_without\_prep | 0.307 | 0.328 | 170.432 | 0.937 | 0.3502 | 0.408 |
| Control - (past) depression | pretest | ielts\_sp\_with\_prep | 0.645 | 0.326 | 167.948 | 1.978 | 0.0496 | 0.856 |
| Control - (past) depression | posttest | ielts\_sp\_with\_prep | 0.345 | 0.328 | 170.432 | 1.052 | 0.2944 | 0.458 |
| Control - (past) depression | pretest | vf\_eng\_total | 0.418 | 0.329 | 171.348 | 1.272 | 0.2049 | 0.555 |
| Control - (past) depression | posttest | vf\_eng\_total | 0.901 | 0.328 | 170.432 | 2.748 | 0.0067 | 1.196 |
| Control - (past) depression | pretest | ENG\_CANDO\_PRODUCTIVE | -0.200 | 0.322 | 161.657 | -0.620 | 0.5358 | -0.265 |
| Control - (past) depression | posttest | ENG\_CANDO\_PRODUCTIVE | -0.082 | 0.319 | 157.727 | -0.256 | 0.7980 | -0.109 |
| Control - (past) depression | pretest | ENG\_CANDO\_RECEPTIVE | -0.032 | 0.319 | 157.794 | -0.101 | 0.9198 | -0.043 |
| Control - (past) depression | posttest | ENG\_CANDO\_RECEPTIVE | 0.018 | 0.319 | 157.727 | 0.056 | 0.9556 | 0.024 |

```
# show significant changes over time
within_group %>% mutate_at(4:7, round,3) %>% mutate_at(8, round,4) %>% mutate_at(9, round,3)  %>% kbl() %>% kable_styling()
```

| contrast | group | variable | estimate | SE | df | t.ratio | p.value | effect.size |
| --- | --- | --- | --- | --- | --- | --- | --- | --- |
| pretest - posttest | Control | ppvt\_raw | -0.410 | 0.293 | 386.310 | -1.399 | 0.1626 | -0.545 |
| pretest - posttest | (past) depression | ppvt\_raw | -0.399 | 0.257 | 386.867 | -1.553 | 0.1213 | -0.529 |
| pretest - posttest | Control | ielts\_ls\_raw | -0.357 | 0.280 | 385.339 | -1.274 | 0.2034 | -0.474 |
| pretest - posttest | (past) depression | ielts\_ls\_raw | -0.774 | 0.294 | 387.042 | -2.631 | 0.0088 | -1.027 |
| pretest - posttest | Control | ielts\_sp\_without\_prep | -0.390 | 0.281 | 385.511 | -1.391 | 0.1649 | -0.518 |
| pretest - posttest | (past) depression | ielts\_sp\_without\_prep | -0.800 | 0.257 | 386.867 | -3.116 | 0.0020 | -1.062 |
| pretest - posttest | Control | ielts\_sp\_with\_prep | -0.380 | 0.281 | 385.511 | -1.353 | 0.1768 | -0.504 |
| pretest - posttest | (past) depression | ielts\_sp\_with\_prep | -0.680 | 0.260 | 387.079 | -2.610 | 0.0094 | -0.902 |
| pretest - posttest | Control | vf\_eng\_total | -1.028 | 0.287 | 385.964 | -3.588 | 0.0004 | -1.364 |
| pretest - posttest | (past) depression | vf\_eng\_total | -0.545 | 0.257 | 386.867 | -2.120 | 0.0346 | -0.723 |
| pretest - posttest | Control | ENG\_CANDO\_PRODUCTIVE | -0.903 | 0.275 | 385.281 | -3.279 | 0.0011 | -1.198 |
| pretest - posttest | (past) depression | ENG\_CANDO\_PRODUCTIVE | -0.785 | 0.248 | 385.847 | -3.161 | 0.0017 | -1.042 |
| pretest - posttest | Control | ENG\_CANDO\_RECEPTIVE | -0.661 | 0.275 | 385.281 | -2.399 | 0.0169 | -0.877 |
| pretest - posttest | (past) depression | ENG\_CANDO\_RECEPTIVE | -0.611 | 0.245 | 385.812 | -2.495 | 0.0130 | -0.811 |

```
# Show at which points a change over time differed between groups
between_groups %>% mutate_at(4:7, round,3) %>% mutate_at(8, round,4) %>% mutate_at(9, round,3) %>% kbl() %>% kable_styling()
```

| time\_pairwise | group\_pairwise | variable | estimate | SE | df | t.ratio | p.value | effect.size |
| --- | --- | --- | --- | --- | --- | --- | --- | --- |
| pretest - posttest | Control - (past) depression | ppvt\_raw | -0.011 | 0.390 | 385.284 | -0.029 | 0.9767 | -0.015 |
| pretest - posttest | Control - (past) depression | ielts\_ls\_raw | 0.417 | 0.406 | 385.043 | 1.026 | 0.3056 | 0.553 |
| pretest - posttest | Control - (past) depression | ielts\_sp\_without\_prep | 0.410 | 0.380 | 384.913 | 1.078 | 0.2816 | 0.544 |
| pretest - posttest | Control - (past) depression | ielts\_sp\_with\_prep | 0.300 | 0.383 | 385.065 | 0.784 | 0.4334 | 0.398 |
| pretest - posttest | Control - (past) depression | vf\_eng\_total | -0.483 | 0.385 | 385.163 | -1.257 | 0.2096 | -0.641 |
| pretest - posttest | Control - (past) depression | ENG\_CANDO\_PRODUCTIVE | -0.118 | 0.370 | 384.059 | -0.318 | 0.7505 | -0.156 |
| pretest - posttest | Control - (past) depression | ENG\_CANDO\_RECEPTIVE | -0.050 | 0.368 | 383.946 | -0.136 | 0.8921 | -0.066 |
