# Supplementary figures and images for: Language Learning as a Non-Pharmacological Intervention in Older Adults with (Past) Depression
Source: Brain Sci. 2025 Sep 15;15(9):991. doi: 10.3390/brainsci15090991 (PMC12468076; doi:10.3390/brainsci15090991)

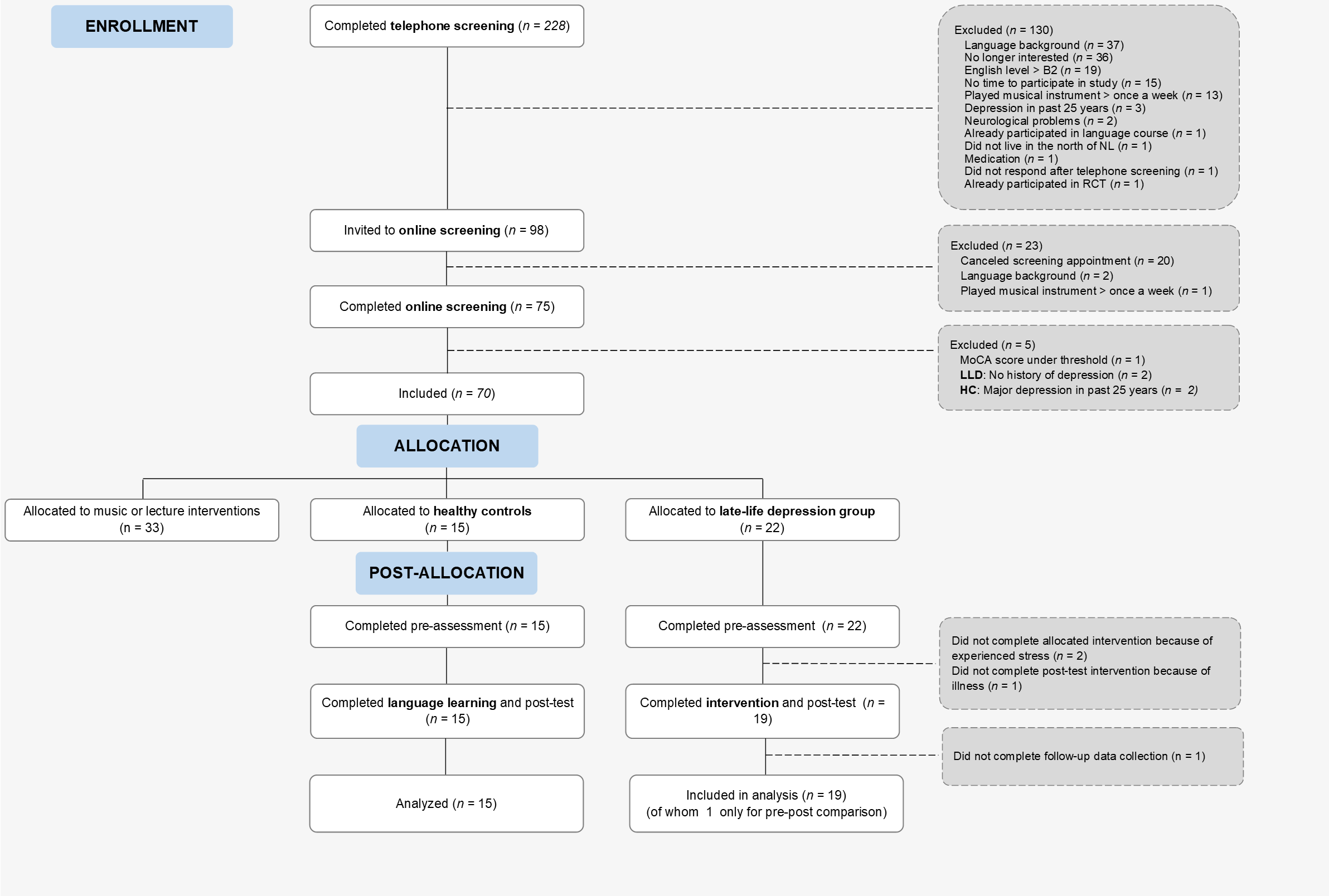

Supplement: Supplementary file 1 [file brainsci-15-00991-s001.zip › S2 - Flowchart for inclusion in the study.png]
